# Short-term transcriptomic response to plasma membrane injury

**DOI:** 10.1038/s41598-021-98420-y

**Published:** 2021-09-27

**Authors:** Swantje Christin Häger, Catarina Dias, Stine Lauritzen Sønder, André Vidas Olsen, Isabelle da Piedade, Anne Sofie Busk Heitmann, Elena Papaleo, Jesper Nylandsted

**Affiliations:** 1 Membrane Integrity, Danish Cancer Society Research Center, Strandboulevarden 49, 2100 Copenhagen, Denmark; 2 Computational Biology Laboratory, Center for Autophagy, Recycling and Disease, Danish Cancer Society Research Center, Strandboulevarden 49, 2100 Copenhagen, Denmark; 3 Translational Disease Systems Biology, Faculty of Health and Medical Sciences, Novo Nordisk Foundation Center for Protein Research University of Copenhagen, Blegdamsvej 3B, 2200 Copenhagen N, Denmark; 4 Department of Cellular and Molecular Medicine, Faculty of Health Sciences, University of Copenhagen, Blegdamsvej 3C, 2200 Copenhagen N, Denmark

**Keywords:** Cell biology, Computational biology and bioinformatics, Genetics, Molecular biology

## Abstract

Plasma membrane repair mechanisms are activated within seconds post-injury to promote rapid membrane resealing in eukaryotic cells and prevent cell death. However, less is known about the regeneration phase that follows and how cells respond to injury in the short-term. Here, we provide a genome-wide study into the mRNA expression profile of MCF-7 breast cancer cells exposed to injury by digitonin, a mild non-ionic detergent that permeabilizes the plasma membrane. We focused on the early transcriptional signature and found a time-dependent increase in the number of differentially expressed (> twofold, *P* < 0.05) genes (34, 114 and 236 genes at 20-, 40- and 60-min post-injury, respectively). Pathway analysis highlighted a robust and gradual three-part transcriptional response: (1) prompt activation of immediate-early response genes, (2) activation of specific MAPK cascades and (3) induction of inflammatory and immune pathways. Therefore, plasma membrane injury triggers a rapid and strong stress and immunogenic response. Our meta-analysis suggests that this is a conserved transcriptome response to plasma membrane injury across different cell and injury types. Taken together, our study shows that injury has profound effects on the transcriptome of wounded cells in the regeneration phase (subsequent to membrane resealing), which is likely to influence cellular status and has been previously overlooked.

## Introduction

It is well established that cells are rapidly able to mount a repair response towards a torn membrane through evolutionary conserved repair mechanisms, thus preventing cell death^5,6^. However, there is a growing appreciation for what happens after initial membrane resealing, when a continuous membrane is re-established (in the sub-second to second range^7–9^). The regeneration phase occurs within minutes to hours^10–13^ and is critical to ensure adequate cellular functioning. In fact, it has been proposed that pathologies may arise not only from defects in membrane resealing but also from the consequences of poor regeneration capacity^13^. We envision that a better understanding of the underlying mechanisms will pave way for the establishment of strategies that can modulate these processes. These strategies would either promote efficient repair in pathologies caused or worsen by poor membrane integrity^11,14^ or inhibit repair in cancer cells to counteract their enhanced membrane repair capacity^15–19^.

It has been reported that the expression profile changes upon plasma membrane (PM) stress^2,3,20,21^. However, only very few studies have analysed the transcriptome after injury and initial repair of the PM in an unbiased and genome-wide manner^1,4,22,23^. These studies tend to focus on long-term expression changes (> 6 days post-injury)^22,23^ and, to our knowledge, no transcriptome analysis has been conducted in the short-term (< 1 h post-injury). Intermediate (few hours) and long-term (24 h) potentiation of membrane resealing at a cellular level, through protein synthesis and gene expression changes respectively, has been suggested as an adaptive response to promote more effective membrane repair^3^. Therefore, understanding which genes and signalling pathways become activated and inhibited is of interest. Ultimately, such knowledge could help to explain the mechanisms that are key for membrane restructuring after initial resealing.

Models of injury have been mainly neuronal^23,24^, renal^22^, cardiac^4^, smooth muscle^21^ and skin^2,3,20,21^, while cancer cells have been largely overlooked. Breast carcinoma cells are of particular interest when investigating cellular changes occurring after wounded cells have resealed their membranes because they are known to be very efficient at recovering from injury, to ensure survival during invasion and metastasis. We have shown that they do this partly by upregulating PM repair proteins^17^, but other injury-induced responses may also contribute.

The aim of this study was to decipher the short-term transcriptomic response related to membrane injury induced by digitonin, a non-ionic saponin that permeabilizes the PM in a cholesterol dependent manner. The two-part experimental design began with the analysis of the transcriptome profile of MCF-7 breast carcinoma cells early during post-injury (20 min, 40 min and 60 min) by RNA-sequencing (RNA-seq). Firstly, we found that immediate-early response genes (IEGs) were among the most upregulated genes across all timepoints and their mRNA expression increased in a time-dependent manner. IEGs are known to be activated and transcribed within minutes to a wide variety of extrinsic stimuli and have essential functions in cellular stress responses including the immune system^25^. Secondly, pathway analysis based on the mRNA changes predicted that from 40 min post-injury, but mainly at 60 min, certain kinase-dependent pathways may be activated, including Mitogen-activated protein kinase (MAPK), Protein kinase C (PKC), Phosphoinositide-3 kinase (PI3K) and Extracellular signal-regulated kinase 5 (ERK5). Thirdly, our analysis also predicted a pronounced activation of multiple inflammation and immune-related pathways, including interleukin (IL) signalling, toll-like-receptor (TLR) signalling and cascades mediated by reactive oxygen species (ROS). The second part of our experimental setup investigated the conserved nature of the 1 h transcriptomic response after PM injury or its immediate downstream effect, Ca^2+^ influx, in other models. Interestingly, our meta-analysis revealed that this three-part transcriptional response to PM injury is conserved^1,4^. The existence of such early and strong immunogenic response was not previously acknowledged.

## Methods

### Cell culture

Human breast carcinoma cells, MCF-7, were cultured at 37 °C and 5% CO_2_ in RPMI 1640 (Gibco) supplemented with 6% heat-inactivated fetal bovine serum (FBS) and 0.25% Penicillin–Streptomycin, which promoted optimal growth conditions.

### Detergent-induced PM wounding experiments

Cholesterol-dependent cytolysins are known to create pores in cellular membranes^26,27^. For example, digitonin is a commonly used membrane pore-forming at cholesterol-rich regions. Because it achieves wide-ranging PM injury, digitonin was used to induce PM wounding. MCF-7 cells were first grown to 70–80% confluency in 10 cm cell culture dishes. Cells were briefly washed with DPBS–Ca^2+^ and then injured by a 10 min incubation with 10–15 µg/ml digitonin, as indicated, diluted in HBSS ± 1.26 mM Ca^2+^ (Gibco). The optimal concentration of digitonin was determined prior to its use in the RNA-seq experiment and was one that causes the majority of cells to die when repair mechanisms are compromised (in the absence of Ca^2+^), while only a minority die when repair mechanisms are competent (under 20% cell death). Therefore, for concentration optimisation experiments, digitonin is incubated in HBSS ± Ca^2+^, while subsequent experiments use HBSS + Ca^2+^ and the absence of digitonin as the negative control.

### Membrane integrity assay

For cell death measurements, after injury cells were incubated with cell-permeable Hoechst-33342 (5 µg/ml) (Sigma-Aldrich) and cell-impermeable propidium iodide (PI) (0.2 µg/ml) (Sigma-Aldrich) diluted in RPMI media. After 5 min, they were imaged using an IX71 microscope coupled to a DP72 camera and Cell^P software (Olympus), followed by automated particle counting using ImageJ. As part of the quantification pipeline, images were subjected to an arbitrary and fixed intensity threshold and binary images were created. Next, the watershed algorithm in ImageJ ran on these images to resolve “touching objects” (i.e. cells in close proximity). Finally, both Hoechst-positive and PI-positive cells were quantified by the particle analysis plugin.

### RNA-seq

At 20-, 40- and 60-min post-injury cells were harvested in TRIzol™ (Invitrogen™) and flash-frozen in liquid nitrogen. Control cells (uninjured) were harvested in the same way. RNA extraction, library construction and transcriptome sequencing were performed by BGI Tech Solutions Hong Kong Co. In total 12 samples (3 samples per condition) were sequenced with 100 bp reads, paired end and at a depth of 20 million reads per sample using Illumina’s HiSeq 4000.

### Bioinformatic analysis

The RNA-seq data was pre-processed before being used for downstream analysis^28^. The pre-processing pipeline included 5 steps: (1) Quality control of the reads was conducted using FastQC, with default settings. (2) Trimmomatic paired end mode was used to trim reads from the 3’ end to remove bases with quality score below 5 and keeping only reads which were longer than 24 bases after trimming. (3) Alignment of the reads to the hg38 reference transcriptome was performed using TopHat2 with the following parameter settings: segment-mismatches = 2, segment-length = 25, and no-coverage-search. (4) Quality control of the aligned reads was conducted using RseQC to get summary statistics about read distribution and transcript coverage distribution. 5) Quantification of reads into integer counts to be used for downstream analysis was performed by using HTSeq with–stranded = no settings.

The downstream analyses included differential expression analysis, gene ontology analysis, pathway enrichment analysis and analysis of upstream pathway regulators. The differential expression analysis was carried out using edgeR^29^ and the quasi-likelihood generalized linear model, after removal of genes expressed at low levels and normalization for library size. We defined significantly expressed genes as genes having FDR < 0.05 and absolute log_2_ fold change > 1. GO analysis was carried out on both the significantly upregulated and downregulated genes (separated analysis) from the expression analysis using the TopGO R package^30^ (*default parameters*). The following parameters are calculated: the total number of genes that are annotated with that specific term is denoted (“Annotated”), the number of significantly differentially expressed genes associated with that term (“Significant”), and the number of genes that would be expected to be significantly associated to each term by random chance (“Expected”). The analysis uses the Fisher’s Exact Test to test the association, and the *P*-values and FDR are calculated.

Pathway enrichment analysis was conducted using ReactomePA^31^ and Ingenuity Pathway Analysis, IPA (Qiagen). Both map different functional pathways to the network of proteins produced by the input genes (related to the mRNA transcripts) based on reference datasets, which for the former is the Ingenuity Knowledge Base^32^ and the latter is the Reactome Knowledge Base^33^. For the analyses using ReactomePA, only DE genes (related to the mRNA transcripts) were considered. This R-package had the p-value and FDR cutoffs set to 0.05. For the analysis using IPA, all genes (related to the mRNA transcripts) were considered along with their relative expression changes and p-values. The cut-off used for the predictive pathway analysis was of − log(*P*-value) > 1.3, which corresponds to *P*-value < 0.05. IPA was not only used for predicting the canonical pathways related to the transcriptomic changes, but also for upstream regulator analyses. The calculated z-scores reflect the predicted activation state of the pathway or potential upstream regulator (a positive/negative z-score indicates activation/inhibition, respectively). While its directionality reflects the predicted activation state, its absolute value predicts the degree of activation/inhibition in a relative manner. This is achieved by comparing transcriptomic changes to molecular networks and literature-derived regulation direction (activation/inhibition)^32^.

All the scripts and processed data related to the bioinformatic analyses are reported in the GitHub repository https://github.com/ELELAB.

## Results

### Plasma membrane injury by digitonin in MCF-7 breast carcinoma cells

**Figure 1:**
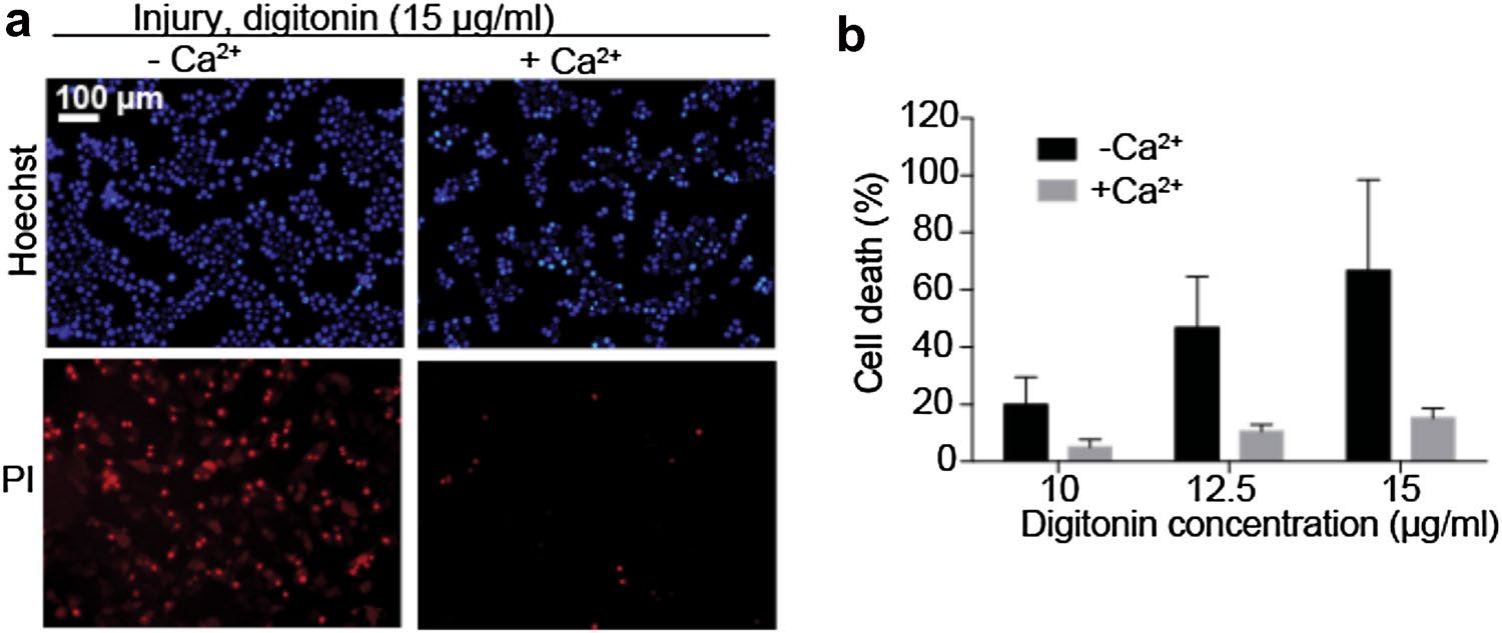
Cells repair from digitonin-induced PM injury in a Ca^2+^-dependent manner. (**a**) Representative images of MCF-7 cells injured by 10 min incubation in 15 µg/ml digitonin in the absence (left panel) or presence (right panel) of Ca^2+^ and then assayed using impermeable PI staining and Hoechst 33,342 (permeable). (**b**) Percentages of PI-positive cells after injury with 10, 12.5 or 15 µg/ml digitonin. Mean and SD of biological triplicates.

Digitonin was used to induce PM injury in MCF-7 breast cancer cells and activates an efficient calcium (Ca^2+^)-dependent repair mechanism to prevent propidium iodide (PI) cellular inclusion (Fig. 1a), in accordance with our earlier findings^6,17,34,35^. To determine a concentration of digitonin that would led to PM damage to a degree that could be repaired, MCF-7 cells were injured in presence or absence of Ca^2+^ for 10 min followed by a 5-min repair period (Fig. 1b). In these experiments PI is not only being used as an indicator of necrotic death, but also a measure of increased membrane permeability (that of the PM and nuclear envelope). All future experiments were conducted using 15 µg/ml of digitonin.

### Transcriptome analysis of MCF-7 cells post-injury

#### Differentially expressed genes

**Figure 2:**
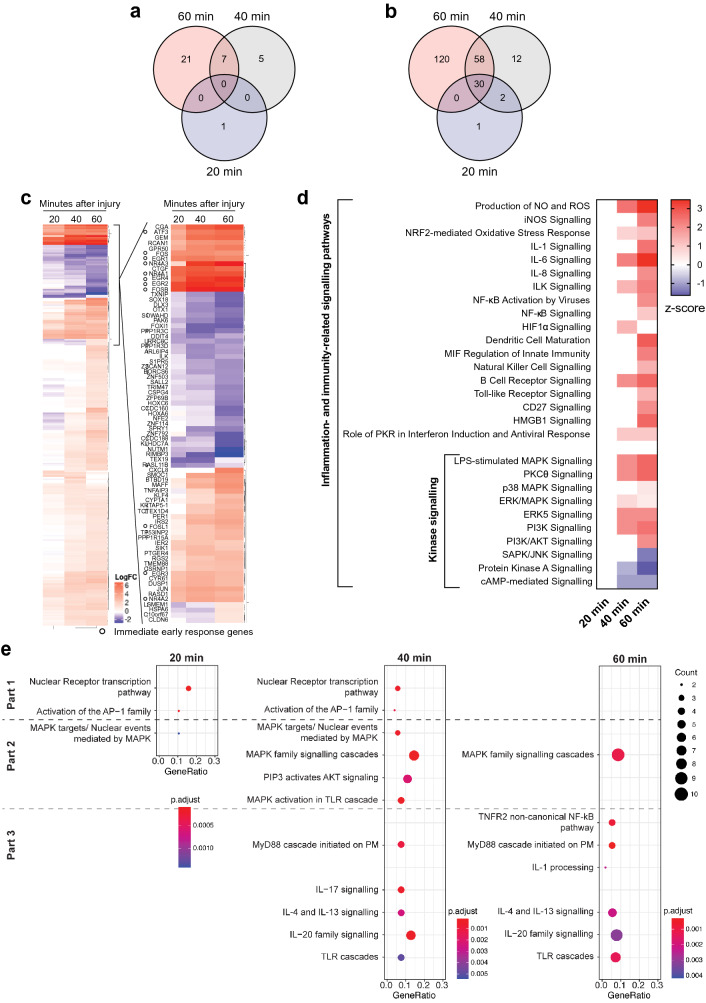
Early injury-induced transcriptional response in MCF-7 breast cancer cells. Overlap of significantly downregulated (**a**) and upregulated (**b**) genes (> twofold, P < 0.05) across the post-injury timepoints assessed (20-, 40- and 60-min post-injury). (**c**) Heatmap depicting the log_2_ fold change of differentially expressed genes (DE) (> twofold, P < 0.05) across the analysed timepoints. The most significantly changed genes are listed and the majority of these show a positive correlation between expression and post-injury time. Round open circles mark immediate-early response genes (IEGs). (**d**) Heatmap showing the signalling cascades predicted by the Ingenuity Pathway Analysis (IPA) to be the most significantly changed at different timepoints post-injury, based on the differentially expressed transcripts (RNA-seq data). These pathways are significantly associated to the dataset (-log(*P*-value) > 1.3) and have a z-score that reflects their predicted activation state (a positive/negative z-score indicates activation/inhibition). Pathways enriched are related to inflammation and immunity and a subset are kinase-dependent. (**e**) Dot-plot showing enriched pathways at 20-, 40- and 60-min post-injury as a function of Gene Ratio (i.e. percentage of total differentially expressed genes in the given GO term), Count (i.e. number of input genes associated to a specific pathway) and *P*-adjusted values. For each timepoint, enriched pathways were grouped into the three parts of the transcriptomic response (*Part 1*: induction of immediate-early response genes; *Part 2:* activation of specific kinase-dependent pathways; *Part 3:* activation of inflammatory and immune responses). Pathway enrichment analysis was conducted by the ReactomePA.

To assess the effect of PM injury at the transcriptional level, MCF-7 cells were treated with a sub-lethal digitonin concentration and the transcriptome was analysed by RNA-seq at 20-, 40- and 60-min post-injury. The number of differentially expressed (DE) mRNA transcripts (fold change ≥ 2 and ≤ -2; *P*-value < 0.05) compared to uninjured cells increased over time, with 33, 102 and 208 mRNA transcripts being significantly upregulated (see Supplementary Table S1 online) and 1, 12 and 28 mRNA transcripts being significantly downregulated (see Supplementary Table S2 online) at 20-, 40- and 60-min, respectively. Although there was no overlap in downregulated DE transcripts across all the timepoints assessed (Fig. 2a), several of the upregulated transcripts were shared (Fig. 2b). The majority of these encode for IEGs, such as early growth response (EGR) 2–4, nuclear receptor subfamily members (NR4A) 1–3, FOS and JUN transcription factors^25^ (Fig. 2c). There is a high correlation between the expression of DE IEGs and post-injury time (Multiple linear regression: R^2^ = 0.9872, *P*-value < 0.05). EGR4, ATF3 and NR4A3 show a positive trend across the timepoints assessed (i.e. its relative mRNA expression increases over time). FOS, on the other hand, despite being also upregulated throughout the post-injury window assessed, peaks at 20 min. The other upregulated IEGs remain largely unchanged across the timepoints assessed. Even when IEGs are excluded, overall the significant transcriptomic changes correlate with post-injury time (Multiple linear regression: R^2^ = 0.9618, *P*-value < 0.05).

#### Gene ontology (GO) analysis and functional pathway analysis

When focusing on significantly associated biological process GO terms, the most enriched were “response to cytokine”, “response to cAMP”, “negative regulation of ERK1 and ERK2 cascades”, “response to lipopolysaccharide”, “cytokine-mediated signalling pathway” and “inactivation of MAPK activity” (see Supplementary Fig. S1a online). As expected, in addition to the “response to cAMP”, other GO terms induced by Ca^2+^- and mechanical-stimulation were also associated (see Supplementary Fig. S1b online). Interestingly, other significantly associated GO terms were related to cellular stress and survival (see Supplementary Fig. S1c online) and both inflammatory and immune responses (see Supplementary Fig. S1d online).

To obtain insight into the functional significance of the GO terms associated to the post-injury transcriptome, we conducted pathway analyses using both the Ingenuity Pathway Analysis (IPA) (Fig. 2d) and ReactomePA (Fig. 2e). The use of multiple platforms is an attempt to counteract knowledge biases (an inherent limitation of this knowledge-guided analysis that has been previously reviewed^36^), achieving a more comprehensive pathway analysis. Together, the GO analysis and pathway analyses performed support a dynamic and gradual three-part transcriptomic response.

The first part of the response, occurring predominantly at 20 min, consists of the activation of transcription factors, such as those that form the AP-1 complex, which influence the expression of stress-related genes (Fig. 2e, *part 1*). At 40 min post-injury, the predominant transcriptomic changes are associated to kinase-dependent cascades (Fig. 2e, *part 2*, and Fig. 2d). These include multiple branches of the MAPK signalling cascade (the canonical ERK, p38 MAPK and the atypical ERK5 – see Supplementary Fig. S2 online^37–39^) and the PKC and PI3K cascades (Fig. 2d).

Overall, the activation of kinase-dependent pathways increases as a function of post-injury time, which is reflected by the increasing z-score (Fig. 2d) and the number of DE genes associated to the pathway (Fig. 2e, *part 2*). Importantly, the induction of kinase-mediated pathways occurred in a specific rather than global manner – evident from the inhibition of the Stress-activated protein kinases (SAPK)/Jun amino-terminal kinases (JNK) branch of MAPK, PKA and cAMP-mediated signalling pathways (Fig. 2d).

The final part of the transcriptomic response is associated to the induction of inflammation- and immunity-related pathways (Fig. 2d,e, *part 3*). Both pathway analyses predicted the activation of IL signalling, TLR cascades and NF-kB-mediated pathways (Fig. 2d,e, *part 3*). In addition, IPA predicted a pronounced activation of redox-mediated pathways, as well as key processes that regulate innate immunity (Fig. 2d).

Although transcriptomic changes at 60-min post-injury can be best characterised by changes in inflammation- and immunity-related pathways, these are activated throughout the response (Fig. 2d). At 40 min particular IL family cascades are significantly associated, namely IL-6 (Fig. 2d), IL-4, IL-13, IL-17 and IL-20 (Fig. 2e, 40 min), while at 60 min post-injury IL-1, IL-6, IL-8 (Fig. 2d), IL-4, IL-13 and IL-20 (Fig. 2e, 60 min) pathways are active. Likewise, ROS-mediated pathways (Fig. 2d) and TLR cascades (Fig. 2d, 40 min and 60 min) are significantly associated to the transcriptome at 40 min and 60 min post-injury, but have an increased *z-score* or count, respectively, at 60 min.

Taken together, the cellular consequences of digitonin-induced PM injury activate a rapid (from 20 min) and sustained (for at least 1 h post-injury) transcriptional response in MCF-7 breast cancer cells that is very dynamic. Initially, the mRNA expression of IEGs is induced and MAPK pathways are predicted to become activated. Later, processes related to cellular stress, inflammation and immunity are activated.

#### Predicted upstream regulators of the transcriptomic response

Our data support a dynamic transcriptomic response to PM injury, both in the nature of the genes expressed and the pathways altered, as well as the magnitude of the transcriptomic changes over time. As a preliminary attempt to understand how these changes could be regulated, we performed an upstream regulator analysis using IPA (see Supplementary Fig. S3 online). Four key transcriptional regulators were found to be activated in a post-injury time-dependent manner (Platelet-derived growth factor (PDGF-BB), Nuclear protein 1 (NUPR1), Tumour necrosis factor (TNF) and ERK) and one inhibited (Histone deacetylases (HDAC)). Of relevance are also G-protein coupled estrogen receptor 1 (GPER1), Triggering receptor expressed on myeloid cells 1 (TREM1), IL1A, IL1B, Signal transducer and activator of transcription 3 (STAT3), Epidermal Growth Factor (EGF) and JUN, which have smaller absolute *z-scores* but also increase in a time-dependent manner.

Only JUN and some members of the TNF family were found to be upregulated in our RNA-seq data. Specifically, JUN recorded log_2_ fold changes of 3.069, 3.614, 3.469 at 20-, 40- and 60-min post-injury, while the transcription of TNF superfamily member 9 (TNFSF9) and TNF alpha induced protein 3 (TNFAIP3) was associated with log_2_ fold changes of 1.365 and 1.590 and 1.661 and 3.878, respectively, at 40- and 60-min, and the TNF receptor superfamily member 11b (TNFRSF11B) of 2.086 at 60-min. Therefore, JUN and TNF family proteins are putative upstream regulators that could explain the observed transcriptomic changes.

### Meta-analysis of the post-injury transcriptome

We next performed a small meta-analysis to better elucidate the transcriptomic response directly or indirectly associated to PM injury. To our knowledge this is the first one in the field and included the only two published studies addressing genome-wide transcriptomic changes following PM injury or PM damage-related events in the short-term (1 h post-injury), along with our dataset. One of the studies analysed was conducted by Wales et al*.*^1^ and assessed the exposure of MCF-7 breast cancer cells to ionomycin, which induces increased cytosolic Ca^2+^ and cytoskeletal remodelling. These are direct cellular consequences of PM injury. The other study was perfomed by Rysä et al*.*^4^ and investigated the response of cardiomyocytes to mechanical stretch, which is known to cause Ca^2+^ influx^40,41^ due to compromised membrane integrity^42–45^. The nature of the meta-analysis not only allows for greater power to detect differential expression and associated pathways^46^, but also questions the conserved nature of the transcriptomic response across different cell and injury types. Furthermore, because Ca^2+^ influx is the common signalling cascade stimulus across all injury types (namely digitonin- and stretch-induced) and “pseudo-injury” (i.e. ionomycin) considered, similarities in transcriptome profile suggest that Ca^2+^ is the driver of the transcriptomic response to PM injury.

#### Differentially expressed genes

**Figure 3﻿:**
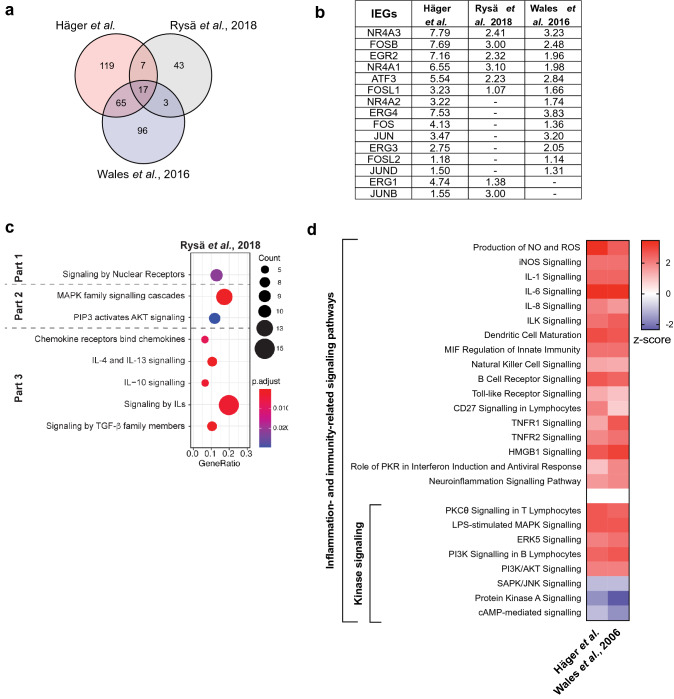
Meta-analysis of the injury-induced transcriptional response (at 1 h post-injury) shows that the transcriptional response is conserved. (**a**) Overlap of significantly upregulated genes (> twofold, P < 0.05) between our RNA-seq dataset derived from digitonin-injured MCF-7 breast cancer cells and two others from ionomycin-treated MCF7 breast cancer cells (Wales et al*.,* 2016^1^) and mechanically stretched cardiomyoctes (Rysä et al*.*, 2018^4^). (**b**) log_2_ fold changes of immediate-early response genes (IEGs) (with P < 0.05) across all datasets included in the meta-analysis. (**c**) Dot-plot showing the predicted enriched pathways from the transcriptomic dataset by Rysä et al*.*^4^ as a function of Gene Ratio (i.e. percentage of total differentially expressed genes in the given GO term), Count (i.e. number of input genes associated to a specific pathway) and *P*-adjusted values. The enriched pathways were grouped into the three parts of the transcriptomic response (*Part 1*: induction of immediate-early response genes; *Part 2:* activation of specific kinase-dependent pathways; *Part 3:* activation of inflammatory and immune responses). Pathway enrichment analysis was conducted by the ReactomePA. (**d**) Heatmap showing the signalling cascades predicted by the Ingenuity Pathway Analysis to be the most significantly changed in our RNA-seq data and the transcriptomic data reported by Wales et al*.,* 2016^1^. These pathways are significantly associated to the dataset (-log(*P*-value) > 1.3) and have a z-score that reflects their predicted activation state (a positive/negative z-score indicates activation/inhibition).

Consistent with our RNA-seq data, the majority of gene expression changes recorded represent upregulation (Fig. 3a) rather than downregulation (see Supplementary Fig. S4a online), when the same cut-off criteria are applied (fold change ≥ 2 and ≤ -2; *P*-value < 0.05). Interestingly, 17 DE mRNA transcripts were consistently upregulated in all studies, including the IEGs EGR2, NR4A 1–3 and FOS (FOSB and FOSL1) (Fig. 3b).

#### GO analysis and functional pathway analysis

Next, we questioned whether the same GO terms would be significantly associated to all three studies (see Supplementary Table S3 online). Indeed, five GO terms were in common. The transcriptome profile of our model was also associated to the same eight GO terms as the model from Wales et al*.*^1^, including GO terms related to calcium signalling, stress responses, kinase signalling and inflammation.

To identify key changes in signalling cascades that could drive the association of these biological processes (GO terms) to the transcriptomic response of all three models in question, we performed pathway analysis using ReactomePA and IPA. As with our digitonin-induced PM injury model, analysis of the transcriptome published by Rysä et al*.*^4^ reveals a response that can be characterised in three parts. Firstly, along with the upregulation of IEGs (Fig. 3b), nuclear-based pathways are upregulated (Fig. 3c, *part 1*). This is expected given that most IEGs are transcription factors. Secondly, specific kinase-dependent cascades are upregulated, namely MAPK (Fig. 3c, *part 2*). Thirdly, multiple inflammatory and immune-related pathways are induced, such as chemokine and cytokine signalling (in particular IL signalling). The heatmap shows that there is great overlap (i.e. similarity) between the transcriptome profile in our model and Rysä et al*.*^4^ (Fig. 3c). Likewise, the transcriptomic dataset presented by Wales et al*.*^1^ also supports the activation of specific kinase-dependent signalling and enrichment of multiple inflammation- and immunity-related signalling pathways (Fig. 3d). Taken together, the activation of IEGs, certain kinase-dependent cascades and inflammation- and immunity-related pathways are common transcriptional events observed in our model and others^1,4^.

#### Predicted upstream regulators of the transcriptomic response

Our meta-analysis identified that all datasets shared largely the same predicted upstream transcriptional regulators (see Supplementary Fig. S4b online). Some of these were predicted to drive the time-dependent transcriptomic changes in our model of injury, namely JUN and TNF (see Supplementary Fig. S3 online). Other predicted upstream transcriptional regulators include transcription factors (e.g. NF-kB^47^), transcription activators (e.g. AP-1 complex^48^), cytokines^49,50^ and TLRs^51^.

Of the predicted upstream regulators in common across all three studies some are upregulated in the RNA-seq of digitonin-injured MCF-7 cells (see Supplementary Fig. S4c online). Some of these regulate in inflammatory and immune responses, by modulating the expression of chemokine receptors^52^, cytokines^53^ and the NF-kB signalling^54,55^. In addition, some of the putative regulators, such as EGR1, are themselves IEGs and/or modulate the expression of IEGs^53,56^, as well as MAPK components^56^. Interestingly, AP1 was the only predicted upstream regulator upregulated in all three studies (see Supplementary Fig. S4c online), thus may be a relevant regulator of the conserved transcriptomic response following the PM injury or its associated downstream effects.

## Discussion

**Figure 4:**
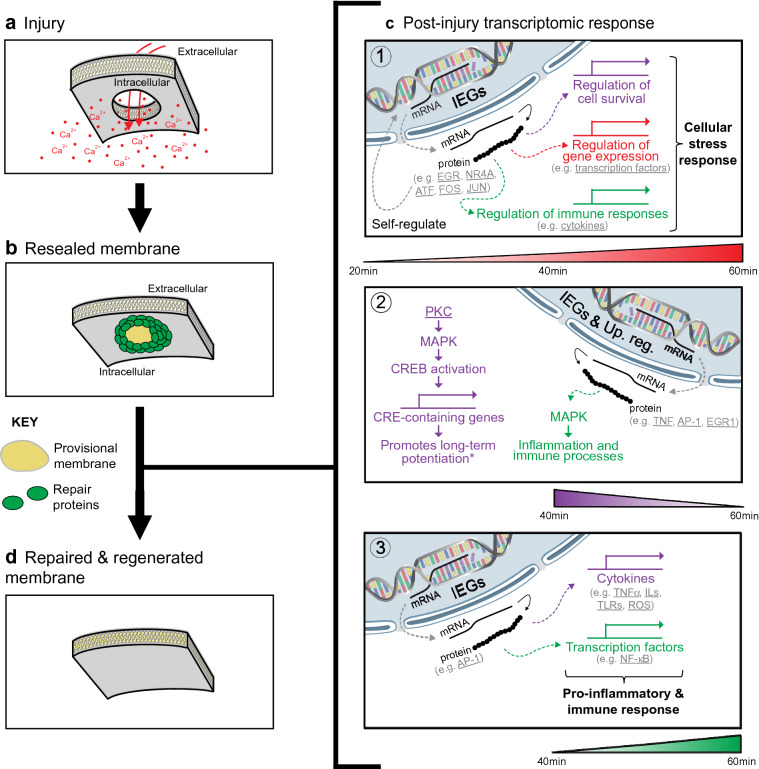
Short-term transcriptomic response following PM injury. (**a**) Injury at the PM results in Ca^2+^ influx. (**b**) Membrane repair mechanisms are rapidly activated and result in immediate PM repair to halt the influx of Ca^2+^ and outflux of intracellular components (e.g. by forming a provisional patch membrane). (**c**) Injury triggers a three-part transcriptomic response: **1.** Activation of immediate-early response genes (IEGs): Increased expression of IEGs regulates the expression of genes involved in the regulation of cell survival, gene expression and immune responses, mounting a cellular stress response. IEGs can self-regulation by inducing their own expression. This initial response dominates the transcriptomic profile at 20 min, although the expression of the majority of IEGs increased as a function of time. **2.** Activation of specific MAPK signalling cascades: Induction of CRE-containing genes may promote long-term potentiation of the membrane for increased resilience to repeated wounding (indicated by the asterisk)^2, 3^. In parallel, increased expression of IEGs and upstream regulators (abbreviated as “Up. reg.”) drives MAPK-mediated signalling, which induces inflammation- and immunity-related processes. **3.** Induction of pro-inflammatory and immune responses: IEGs induce the expression of cytokines and transcription factors that result in a pro-inflammatory milieu and drive a strong immune response. (**d**) Ultimately, the PM fully repairs and is regenerated to adopt its original composition and properties. We hypothesise that this post-injury transcriptional response is key for adequate membrane repair and regeneration, restoring plasma membrane functionality. Genes, proteins and protein families underlined were found to be changed in the RNA-seq data, while pathways and transcriptional events mentioned that are not underlined were predicted from the RNA-seq data (except for that marked with an asterisk).

Disruptions to the PM trigger a robust repair response that is predominantly driven by the abrupt influx of Ca^2+^. While the first phase of the response aims to physically re-seal the PM, less is known about the secondary regeneration phase that follows. Here, we show that PM injury induced a strong transcriptional response in MCF-7 breast cancer cells (summarised in Fig. 4). This response is gradual and dynamic, involving (1) the activation of IEGs, (2) the activation of specific MAPK cascades and (3) the induction of inflammatory and immune pathways.

Our meta-analysis investigating transcriptional changes in response to PM injury and/or its associated cellular consequences (such as Ca^2+^ influx) also supported this three-part response. In this comparative approach, the DE genes identified in our dataset (derived from MCF-7 cells injured with digitonin) were compared with those identified from MCF-7 cells treated with ionomycin (Wales et al*.*^1^) and cardiomyocytes injured by mechanical stretching (Rysä et al*.*^4^). To our knowledge, these are the only unbiased, genome-wide study looking at short-term transcriptomic changes. When analysed in parallel to our transcriptomic data, they aid in the understanding of what changes could be driven by the directly related calcium influx, which is an inevitable cellular consequence of PM injury^57^. This is because Wales and colleagues^1^ used a pharmacological approach to increased cytosolic Ca^2+^ (acting as a “pseudo” injury), while Rysä et al*.*^4^ exposed a different cell line to mechanical stretching, which also results in increased Ca^2+^ influx^40,41^ due to compromised membrane integrity^42–45^.

Our findings add PM injury to the established list of broad stimuli^25^ that activate IEGs and our time-course analysis suggests that this transcriptional response occurs within minutes, being significantly detected at 20-min post-injury. Specifically, the mRNA expression of the nuclear receptors NR4A 1–3 and the transcription factors EGR 2–4, FOS and JUN are induced (Fig. 2b). Most of these were also induced upon membrane stress^4^ and cytosolic Ca^2+^ influx^1^ (Fig. 3b). Although this upregulation was previously reported by Rysä and colleagues^4^, our time-course analysis of wounded MCF-7 cells and our meta-analysis validate and support the conserved nature of the transcriptomic response across different cell and injury types.

IEGs regulate the expression of target genes involved in the regulation of gene expression^24,25,48,58,59^, cell survival^58,59^ and immune responses^24,25,48^. These activities place the AP-1 protein complex (encoded by FOS and JUN), as well as other IEGs^25^, at a key position in the regulation of cellular stress responses. It is therefore not surprising that the induction of IEG expression is a prevailing part of the transcriptomic response to PM injury and its associated cellular consequences. Likewise, IEGs have been found by others to be induced at 1 h post-injury^20^, as well as days after injury^23^. Moreover, it has been proposed that c-fos gene expression (and possibly other IEGs too) is proportional to the degree of PM disruption (due to its Ca^2+^-responsive element), influencing the activation of its target genes^21^. Wales et al*.*^1^ also found IEGs to be part of a cohort of genes directly regulated by Ca^2+^, through the phenomenon of Ca^2+^-mediated actin reset (i.e. the sudden shift of actin distribution in response to increased intracellular Ca^2+^)^1^. Given that PM injury also results in pronounced Ca^2+^ influx, this phenomenon is likely to modulate, at least in part, the post-injury transcriptomic response.

Beyond functioning as a notable activator of signalling cascades (e.g. activating PM repair proteins^60^), abrupt Ca^2+^ influx imposes some degree of cytotoxicity^61^. The AP-1 complex is also known to directly influence the balance between cell death and survival^58^, although the end-effect is influenced by cell type, cellular status, expression levels and the composition of the complexes^62,63^. The literature is controversial and c-jun has been associated with both cell death^64,65^ and cell survival^59^ in tumours. In MCF-7, specifically, the literature favours its pro-survival activity^66^, as does transient induction of IEGs compared to chronic^58^. Indeed, “cell stress and survival” was one of the most associated GO terms (see Supplementary Fig. S1c online) and JNK signalling, which is believed to be important in initiating apoptosis^67^, was predicted to be downregulated (Fig. 2d). Also, within the post-injury window investigated, we did not observe the induction of pro-apoptotic genes in digitonin-induced MCF-7 cells subsequent to the early expression of IEGs. Therefore, the induction of IEGs may function to promote cell survival in response to PM injury (in cells that were able to reseal their membranes), which is hypothesised to occur frequently during cancer metastasis and invasion^17^.

Beyond dictating cellular fate, IEGs might ensure that an adequate cellular response is mounted towards injury. This would be advantageous for cells for multiple reasons. Firstly, by being regulated by Ca^2+^, its expression/activity can be proportional to the degree of injury. Secondly, IEG induction is hypothesised to occur early in the repair response (detected at 20 min post-injury) and IEGs are capable of driving the transcriptomic response that follows. In fact, IEGs were predicted to be key upstream regulators of the transcriptomic response (see Supplementary Fig. S3, S4b and S4c online). Moreover, other predicted upstream regulators may also regulate IEG expression, namely PDGF-BB^68^, NUPR1^69^, TNF^53^, ERK^70^, HDAC^71^, TREM1^72^, IL1B^73^, STAT3^74^, EGF^75^ and JUN^76^. Two other factors that would favour an advantage regulatory role of IEGs in the response to injury is the fact that they self-regulate the expression of each other^77^ and seem to be conserved across different cell and injury types. Therefore, IEGs are likely to govern major aspects of the injury response—a concept that was previously proposed^24,25^ but not linked to PM injury.

A predominant part of the transcriptomic response that we characterize here is the robust activation of cascades involved in inflammation and immunity. Both IEGs^48,77^ and kinase-dependent pathways^78–85^ have been suggested to play a key role in inflammatory. The AP-1 complex can, for example, directly regulate the expression of cytokines (including TNF-α and ILs) and transcription factors (e.g. NF-κB)^48,77,86^. Other predicted upstream regulators that could regulate inflammatory and immune responses are NUPR1^69^, TNF^53^, ERK^87^, TREM1^88^, IL1A^89^, IL1B^73^, STAT3^74^ and EGF^90^. Some of these exert this effect through MAPK-mediated signalling (e.g. TNF^53^) or through their intrinsic kinase activity^91^. Interestingly, as with the expression of IEGs, Ca^2+^-mediated actin reset may also regulate the expression of inflammatory genes^1^.

We predict that the transcriptional response mounted upon PM injury is mainly pro-inflammatory, as the majority of the activated pathways are pro-inflammatory^47,92^ (involving TLR, IL-1, IL-8, TNF, NF-kB, iNOS and ROS). TLRs are known to mediate innate immunity in response to a wide variety of pathogenic threats through recognition of conserved pathogen-associated molecular motifs^93^, although PM injury has not been previously added to that list. TLRs and proinflammatory cytokines activate the canonical pathway of NF-kB signalling, which regulates the expression of proinflammatory and cell survival genes. Furthermore, through its canonical and alternative pathways (identified in Fig. 2d,e 60 min respectively), NF-kB signalling mediates both innate and adaptive immunity^47,94^. This pro-inflammatory and immunogenic profile arise at 40 min post-injury (Fig. 2d,e) and becomes gradually activated throughout the response. Likewise, transcriptomic data from Rysä et al*.*^4^ and Wales et al*.*^1^ also predicted a strong immunogenic response using two independent pathway analysis software (Fig. 3c,d).

An injury-induced inflammatory and immunogenic response has not been given much focus in the literature. However, there is some evidence to suggest its existence, namely the activation of NF-kB upon PM disruption^21^ and the upregulation of cytokines (including TGF-β) due to PM stress^22^. These events are believed to trigger the expression of stress-induced genes and promote wound-healing^21,22^. This supports our hypothesis that the immediate-early response and the inflammatory and immune responses are key in the transcriptomic response post-injury. However, it remains to be determined the functional significance of the pro-inflammation and immunogenic response post-injury. Whether it contributes the phenomenon of long-term potentiation^3^ to improve repair, or is an intrinsic part of membrane regeneration following injury (resembling inflammation and remodelling in wound healing^95^), remains to be answered.

MAPK signalling (the canonical ERK, p38 MAPK and the atypical ERK5) is known to response to a diverse array of stress stimuli^96^. Moreover, the activation of specific MAPK cascades following injury has also been previously given some degree of attention^2–4,20,22,23^. Likewise, our findings support the gradual activation of specific MAPK cascades clearly from 40 min post-injury (Fig. 2d,e), but possibly as early as 20 min (Fig. 2e, *part 1*), and across different models (Fig. 3d). PKA activation, for example, has been implicated in membrane resealing kinetics^97^ and early potentiation of Ca^2+^-regulated exocytosis to facilitate subsequent wound resealing^3^. However, our time-course analysis and meta-analysis predicted that PKA and cAMP-mediated signalling pathways are inhibited (Figs. 2d, 3d). This discrepancy may be due to cell type differences (the studies referenced were performed in neurons and fibroblasts) or, more likely, differences in post-injury times assayed. PKA activation was detected early post-injury in the study (< 5 min)^97^, thus the predicted inactivation of PKA and cAMP-mediated signalling pathways observed in our study may reflect a regulatory negative feedback loop, which would be expected following acute activation. At a later stage post-injury, PKC was reported to mediate cAMP response element-binding protein (CREB) activation via a p38 MAPK pathway^2^ to induce expression of cAMP-response element (CRE)-containing genes and promote long-term potentiation^2,3^. Likewise, our predictive pathway analyses predicted the activation of MAPK- and PKC-mediated signalling cascades (Figs. 2d, 3d) and CREB1 and p38 MAPK are predicted activated upstream regulators (see Supplementary Fig. S3 and S4 online). Therefore, these specific kinase-dependent cascades may also become activated in our study to promote long-term potentiation, improving the regenerative ability of cells. Although this requires further investigation, modulating kinase-dependent pathways to facilitate regeneration could have clinical significance.

Although the existence of injury-induced changes in gene expression has been previously hypothesised^3,21^ and investigated in a gene-specific manner^20,21^, the field lacked evidence regarding the temporal-specificity of global transcriptomic changes. In particular, short-term (< 1 h) transcriptomic responses were not previously investigated, as opposed to long-term changes (> 6 h)^22–24^. Our study is therefore a step towards deciphering these, although the findings presented would benefit from further validation. This should include evaluation of the transcriptome profile using an additional and independent method and assessment of whether transcriptional changes translate to protein levels and changes in the predicted pathways. Once validated, functional studies should assess the individual and cumulative roles of the three distinct parts of the transcriptomic response (i.e. activation of IEGs, activation of MAPK cascades and induction of inflammation and immune-related processes) on post-injury cellular dynamics. Furthermore, it would be also interesting to understand whether the injury-induced transcriptome is driven as a defensive response to acute trauma and as an adaptive response to potentiate membrane resealing for subsequent injuries. This would not only be significant within the field of membrane repair, but also within cell death and survival, as injury and repair processes are likely to be intimately related.

We show that MCF-7 cancer cells exposed to digitonin-induced membrane injury respond, at a transcriptional level, by expressing IEGs, activating specific kinase-dependent cascades and mounting a robust pro-inflammatory and immunogenic response. Our meta-analysis demonstrated that this three-part response may be conserved across different cell types and injuries, although this should be addressed further.

## Supplementary Information

Supplementary Information.

## Abbreviations

PM: Plasma membrane
IEGs: Immediate-early response genes
MAPK: Mitogen-activated protein kinase
PKC: Protein kinase C
PI3K: Phosphoinositide-3 kinase
ERK5: Extracellular signal-regulated kinase 5
PKA: Protein kinase A
IL: Interleukin
TLR: Toll-like-receptor
ROS: Reactive oxygen species
FBS: Fetal bovine serum
PI: Propidium iodide
EGR: Early growth response
NR4A: Nuclear receptor subfamily members
GO: Gene ontology
IPA: Ingenuity Pathway Analysis
SAPK: Stress-activated protein kinases
JNK: Jun amino-terminal kinases
PDGF-BB: Platelet-derived growth factor
NUPR1: Nuclear protein 1
TNF: Tumour necrosis factor
HDAC: Histone deacetylases
GPER1: G-protein coupled estrogen receptor 1
TREM1: Triggering receptor expressed on myeloid cells 1
STAT3: Signal transducer and activator of transcription 3
EGF: Epidermal Growth Factor
TNFSF9: TNF superfamily member 9
TNFAIP3: TNF alpha induced protein 3
TNFRSF11B: TNF receptor superfamily member 11b
TIAF1: TGFB1-induced anti-apoptotic factor 1
CREB: CAMP response element-binding protein
CREBR: CREB3 regulator factor
CRE: CAMP-response element
