# Supplementary material for: Short-term transcriptomic response to plasma membrane injury
Source: Sci Rep. 2021 Sep 27;11:19141. doi: 10.1038/s41598-021-98420-y (PMC8476590; doi:10.1038/s41598-021-98420-y)
Supplement: Supplementary file 1 — Supplementary Information. [file 41598_2021_98420_MOESM1_ESM.pdf]

Supplementary Information

Short-term transcriptomic response to plasma membrane injury

Swantje Christin Häger[1,5], Catarina Dias[1,5], Stine Lauritzen Sønder[1], André Vidas Olsen[2], Isabelle da Piedade[2], Anne Sofie Busk Heitmann[1], Elena Papaleo[2,3] & Jesper Nylandsted[1,4,✉]

[1]Membrane Integrity, Danish Cancer Society Research Center, Strandboulevarden 49, 2100 Copenhagen, Denmark. [2]Computational Biology Laboratory, Center for Autophagy, Recycling and Disease, Danish Cancer Society Research Center, Strandboulevarden 49, 2100 Copenhagen, Denmark. [3]Translational Disease Systems Biology, Faculty of Health and Medical Sciences, Novo Nordisk Foundation Center for Protein Research University of Copenhagen, Blegdamsvej 3B, 2200 Copenhagen N, Denmark. [4]Department of Cellular and Molecular Medicine, Faculty of Health Sciences, University of Copenhagen, Blegdamsvej 3C, 2200 Copenhagen N, Denmark. [5]These authors contributed equally: Swantje Christin Häger and Catarina Dias. ✉email: jnl@cancer.dk

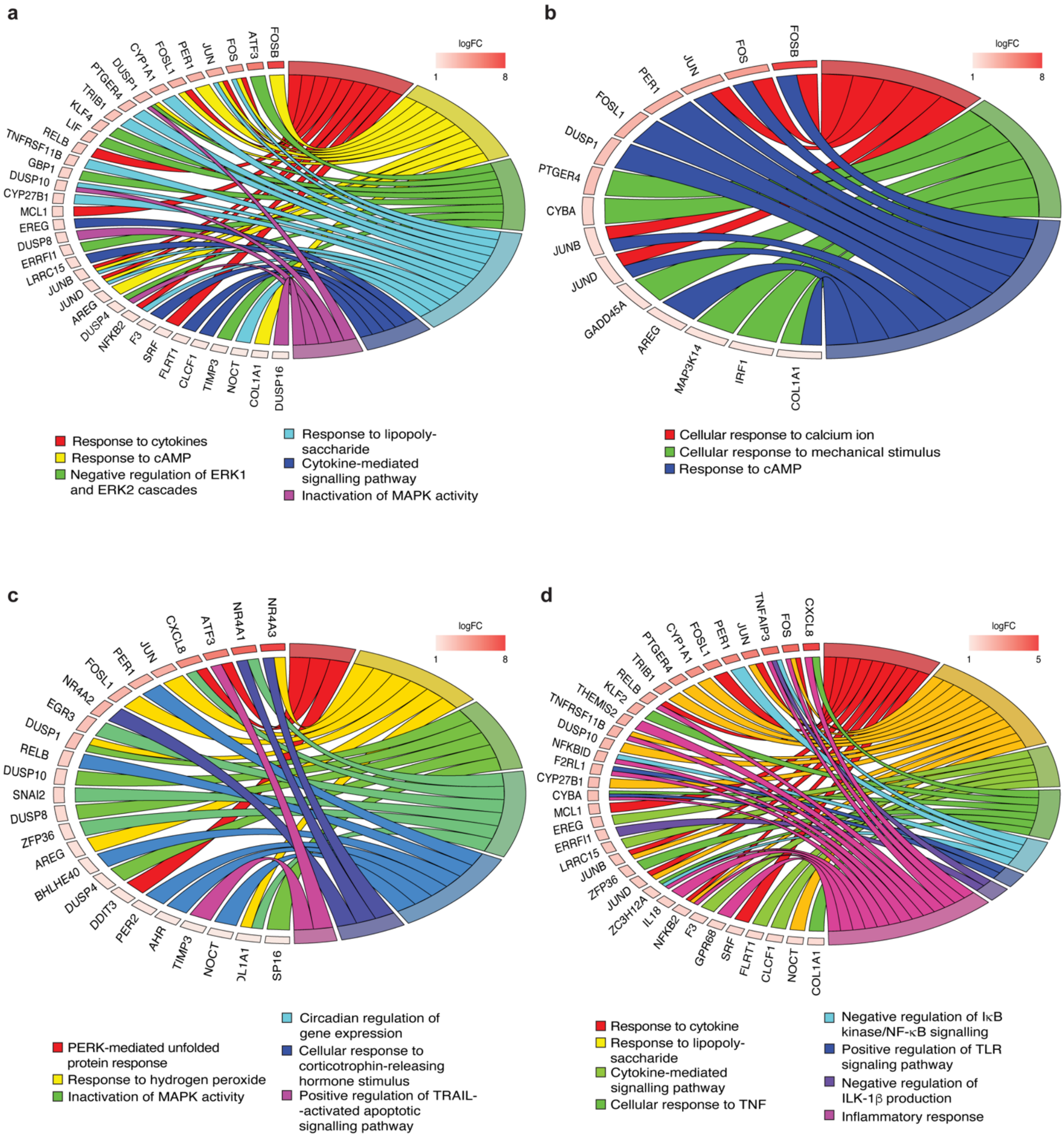

Supplementary figure S1: GO terms related to the injury-induced transcriptional response
Circular plots of the GO terms significantly associated to the RNA-seq data obtained 60 min post-injury, with the corresponding regulated genes and their log_2 fold change. The most enriched (**a**), Ca^{2+}-regulated (**b**), stress-related (**c**) and inflammatory and immune-related (**d**) GO terms are represented.

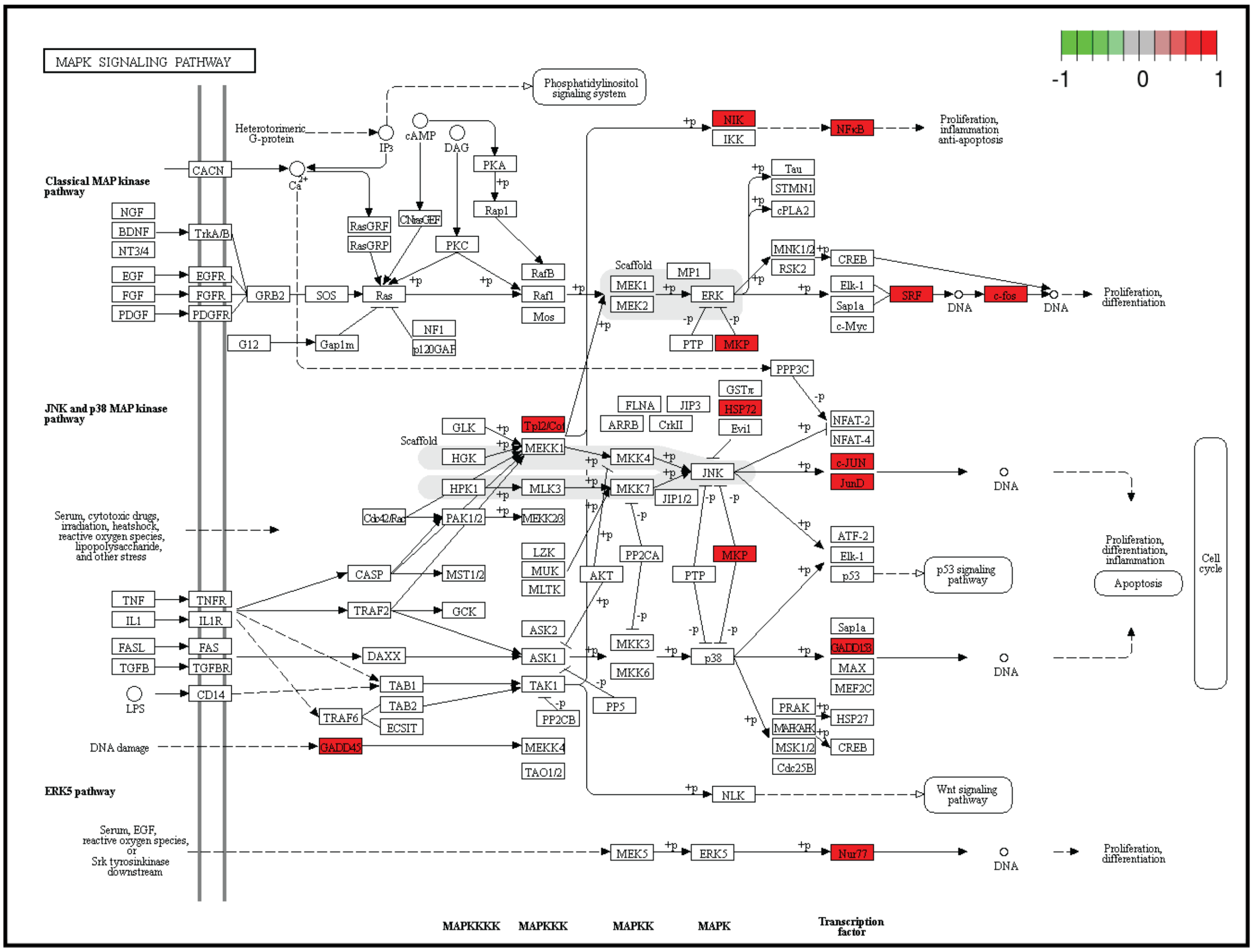

Supplementary figure S2: Predicted regulation of the MAPK signalling cascade at 60 min post-injury in digitonin-injured MCF-7 breast cancer cells

KEGG pathway[37-39] graph rendered by Pathview showing the different branches of the MAPK signalling cascade (the canonical ERK, p38 and JNK and the atypical ERK5) with colour-coded protein symbols to represent increased mRNA expression (in red) detected in the dataset. These are encoded by the following gene symbols: NIK (MAP3K14), NF-kB (NFKB), SRF (SRF), c-fos (FOS), MKP (DUSP1), Tpl2/Co1 (MAP3K8), HSP72 (HSPA6), c-JUN (JUN), JunD (JUND), GADD153 (DDIT3), GADD45 (GADD45A) and Nur77 (NR4A1).

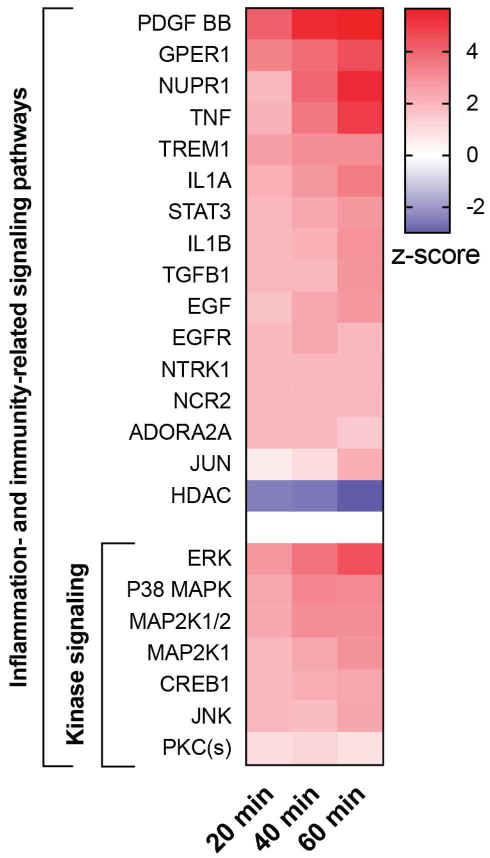

Supplementary figure S3: Potential upstream regulators of the post-injury transcriptomic response
Potential upstream transcriptional regulators responsible for the mRNA changes detected for all post-injury timepoints analysed (predicted by the Ingenuity Pathway Analysis). These upstream transcriptional regulators are significantly associated to the dataset (-log(*p*-value) > 1.3) and have a z-score that reflects their predicted activation state (a positive/negative z-score indicates activation/ inhibition) – plotted in the heatmap.

a

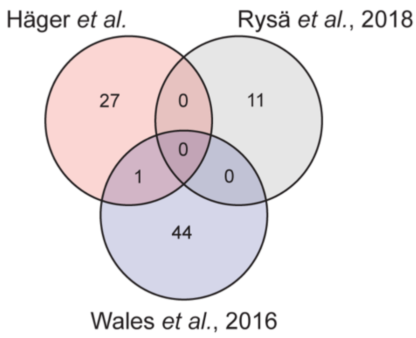

c

Gene symbol	Gene name	Häger et al.	Rysä et al., 2018	Wales et al., 2016
CREBRF	CREB3 regulatory factor	1.406	-	-
CGA	Glycoprotein hormones	6.760	-	1.83
NF-kB inhibitor				
NFKBID	NFKB inhibitor delta	1.894	-	1.42
NFKBIA	NFKB inhibitor alpha	1.632	-	1.58
NFKBIZ	NFKB inhibitor zeta	-	0.850	0.95
TGFB1-induced anti-apoptotic factor 1				
TIAF1	TGFB1-induced anti-apoptotic factor 1	1.170	-	-
EGR1	Early growth response 1	4.743	1.379	0.67
AP-1				
JUN	Jun proto-oncogene	3.469	0.848	3.20
JUNB	JunB proto-oncogene	1.552	1.070	0.95
JUND	JunD proto-oncogene	1.495	-	1.31
FOSB	FosB proto-oncogene	7.689	3.000	2.48
FOS	Fos proto-oncogene	4.131	-	1.36
FOSL1	FOS like 1	3.233	2.233	1.66
FOSL2	FOS like 2	1.814	0.766	1.14

b

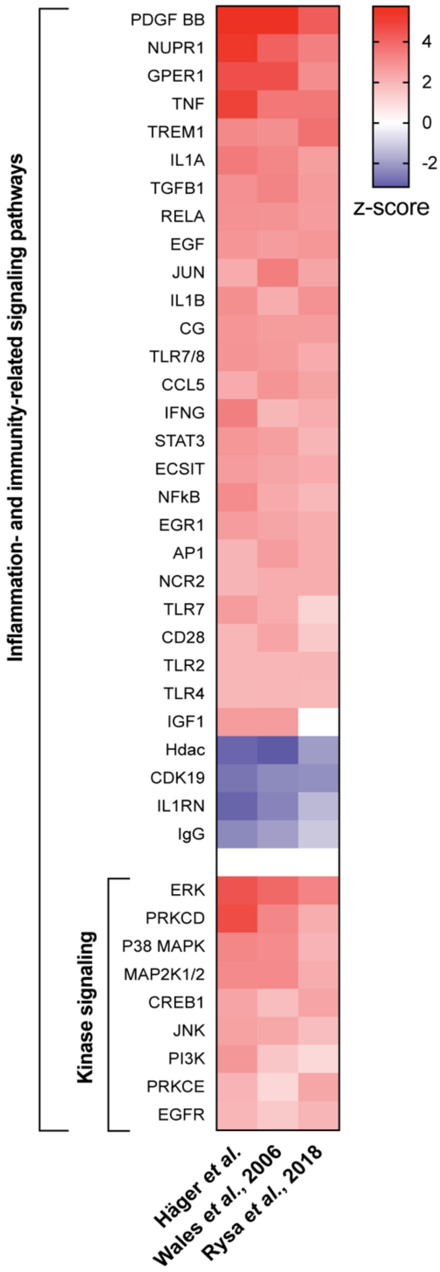

Supplementary figure S4: Meta-analysis of potential upstream regulators of the transcriptomic response

a) Overlap of significantly downregulated genes (>2-fold, P < 0.05) between our RNA-seq dataset derived from digitonin-injured MCF-7 breast cancer cells and two others from ionomycin-treated MCF7 breast cancer cells (Wales *et al.,* 2016) and mechanically stretched cardiomyoctes (Rysä *et al.*, 2018).

b) Potential upstream transcriptional regulators responsible for the mRNA changes detected for the three post-injury RNA-seq datasets (predicted by the Ingenuity Pathway Analysis). These upstream transcriptional regulators are significantly associated to the dataset (-log(*p*-value) > 1.3) and have a z-score that reflects their predicted activation state (a positive/negative z-score indicates activation/inhibition) – plotted in the heatmap.

c) Log_2 fold changes of the predicted upstream regulators (with P < 0.05) that were identified across the datasets included in the meta-analysis.

Supplementary Table S1: List of significantly upregulated differentially expressed genes obtained from RNA-sequencing of digitonin-injured MCF-7 breast cancer cells at 20-, 40- and 60-min post-injury.

20 min timepoint

No.	Ensemble ID	Gene	Full gene name	logFC	F	P value	FDR
1	ENSG00000120738	EGR1	Early growth response 1	4.842	9989.66	3.30E-25	4.31E-21
2	ENSG00000170345	FOS	Fos proto-oncogene	4.408	7190.79	5.54E-24	3.61E-20
3	ENSG00000123358	NR4A1	Nuclear receptor subfamily 4 group A member 1	5.865	6542.85	1.24E-23	5.41E-20
4	ENSG00000177606	JUN	Jun proto-oncogene	3.069	3188.95	5.85E-21	1.91E-17
5	ENSG00000179388	EGR3	Early growth response 3	2.522	3090.91	7.64E-21	1.99E-17
6	ENSG00000125740	FOSB	FosB proto-oncogene	6.784	2490.39	4.84E-20	1.05E-16
7	ENSG00000160888	IER2	Immediate early response 2	2.130	2091.31	2.15E-19	3.89E-16
8	ENSG00000153234	NR4A2	Nuclear receptor subfamily 4 group A member 2	2.734	2065.22	2.39E-19	3.89E-16
9	ENSG00000122877	EGR2	Early growth response 2	6.749	1533.83	3.01E-18	4.36E-15
10	ENSG00000062716	VMP1	Vacuole membrane protein 1	1.372	1420.08	5.79E-18	7.54E-15
11	ENSG00000108551	RASD1	Ras related dexamethasone induced 1	2.837	1324.95	1.04E-17	1.24E-14
12	ENSG00000120129	DUSP1	Dual specificity phosphatase 1	2.718	1051.65	7.39E-17	8.03E-14
13	ENSG00000162772	ATF3	Activating transcription factor 3	3.201	809.00	6.76E-16	6.79E-13
14	ENSG00000171223	JUNB	JunB proto-oncogene	1.477	776.56	9.55E-16	8.89E-13
15	ENSG00000128016	ZFP36	ZFP36 ring finger protein	1.457	732.32	1.56E-15	1.36E-12
16	ENSG00000142871	CYR61	Cysteine rich angiogenic inducer 61	2.613	661.73	3.66E-15	2.98E-12
17	ENSG00000159200	RCAN1	Regulator of calcineurin 1	2.870	516.11	2.92E-14	2.12E-11
18	ENSG00000118523	CTGF	Connective tissue growth factor	5.831	465.70	6.87E-14	4.72E-11
19	ENSG00000144655	CSRNP1	Cysteine and serine rich nuclear protein 1	1.335	448.20	9.45E-14	6.16E-11
20	ENSG00000135625	EGR4	Early growth response 4	5.488	444.49	1.01E-13	6.28E-11
21	ENSG00000164949	GEM	GTP binding protein overexpressed in skeletal muscle	3.510	377.14	3.93E-13	2.33E-10
22	ENSG00000130513	GDF15	Growth differentiation factor 15	1.285	245.86	1.29E-11	7.00E-09
23	ENSG00000179094	PER1	Period circadian clock 1	1.121	102.61	1.15E-08	4.18E-06
24	ENSG00000162891	IL20	Interleukin 20	1.387	91.82	2.61E-08	8.52E-06
25	ENSG00000119508	NR4A3	Nuclear receptor subfamily 4 group A member 3	1.612	83.36	5.27E-08	1.56E-05
26	ENSG00000135346	CGA	Glycoprotein hormones	3.933	61.79	4.31E-07	0.000102455
27	ENSG00000105219	CNTD2	Cyclin N-terminal domain containing 2	1.330	23.97	0.000132207	0.014365355
28	ENSG00000186352	ANKRD37	Ankyrin repeat domain 37	1.112	23.88	0.000134938	0.014540965
29	ENSG00000102195	GPR50	G protein-coupled receptor 50	3.274	23.68	0.000140998	0.015069471
30	ENSG00000172803	SNX32	Sorting nexin 32	1.740	23.25	0.000154656	0.016262597
31	ENSG00000112309	B3GAT2	Beta-1	1.731	21.37	0.000236688	0.02173359
32	ENSG00000142178	SIK1	Salt inducible kinase 1	2.188	18.59	0.000462708	0.038185087
33	ENSG00000139278	GLIPR1	GLI pathogenesis related 1	1.020	17.18	0.000664156	0.048110741

40 min timepoint

No.	Ensemble ID	Gene	Full gene name	logFC	F	P value	FDR
1	ENSG00000125740	FOSB	FosB proto-oncogene	7.718	3999.24	2.34E-23	3.38E-20
2	ENSG00000122877	EGR2	Early growth response 2	7.446	2561.61	1.50E-21	1.63E-18
3	ENSG00000119508	NR4A3	Nuclear receptor subfamily 4 group A member 3	7.217	6559.82	2.28E-25	9.86E-22
4	ENSG00000135625	EGR4	Early growth response 4	7.087	1509.06	2.07E-19	1.42E-16
5	ENSG00000123358	NR4A1	Nuclear receptor subfamily 4 group A member 1	6.595	8987.74	1.19E-26	1.11E-22

No.	Ensemble ID	Gene	Full gene name	logFC	F	P value	FDR
6	ENSG00000118523	CTGF	Connective tissue growth factor	6.288	1693.19	7.10E-20	6.15E-17
7	ENSG00000135346	CGA	Glycoprotein hormones	6.141	369.92	8.09E-14	2.10E-11
8	ENSG00000164949	GEM	GTP binding protein overexpressed in skeletal muscle	5.578	1374.09	4.94E-19	3.06E-16
9	ENSG00000120738	EGR1	Early growth response 1	5.099	8648.81	1.71E-26	1.11E-22
10	ENSG00000162772	ATF3	Activating transcription factor 3	5.082	4840.52	3.92E-24	7.28E-21
11	ENSG00000102195	GPR50	G protein-coupled receptor 50	4.647	85.81	1.96E-08	1.76E-06
12	ENSG00000170345	FOS	Fos proto-oncogene	4.459	6361.50	3.04E-25	9.86E-22
13	ENSG00000159200	RCAN1	Regulator of calcineurin 1	4.235	3445.61	9.43E-23	1.22E-19
14	ENSG00000177606	JUN	Jun proto-oncogene	3.614	4686.00	5.31E-24	8.63E-21
15	ENSG00000198732	SMOC1	SPARC related modular calcium binding 1	3.592	106.02	3.68E-09	3.62E-07
16	ENSG00000153234	NR4A2	Nuclear receptor subfamily 4 group A member 2	3.501	5016.79	2.81E-24	6.47E-21
17	ENSG00000108551	RASD1	Ras related dexamethasone induced 1	3.386	2001.99	1.49E-20	1.49E-17
18	ENSG00000185022	MAFF	MAF bZIP transcription factor F	3.248	732.69	1.64E-16	6.44E-14
19	ENSG00000120129	DUSP1	Dual specificity phosphatase 1	3.172	1711.36	6.43E-20	5.97E-17
20	ENSG00000142871	CYR61	Cysteine rich angiogenic inducer 61	3.172	1639.22	9.60E-20	7.34E-17
21	ENSG00000185950	IRS2	Insulin receptor substrate 2	3.166	960.69	1.35E-17	6.51E-15
22	ENSG00000179388	EGR3	Early growth response 3	3.093	4982.99	2.99E-24	6.47E-21
23	ENSG00000171522	PTGER4	Prostaglandin E receptor 4	2.886	72.90	6.86E-08	5.34E-06
24	ENSG00000222009	BTBD19	BTB domain containing 19	2.886	200.28	1.80E-11	3.34E-09
25	ENSG00000179094	PER1	Period circadian clock 1	2.812	1074.14	4.83E-18	2.51E-15
26	ENSG00000142178	SIK1	Salt inducible kinase 1	2.763	26.92	5.41E-05	0.001654033
27	ENSG00000167874	TMEM88	Transmembrane protein 88	2.482	144.34	2.92E-10	3.68E-08
28	ENSG00000175592	FOSL1	FOS like 1	2.390	185.12	3.54E-11	5.98E-09
29	ENSG00000144655	CSRNP1	Cysteine and serine rich nuclear protein 1	2.389	1646.61	9.21E-20	7.34E-17
30	ENSG00000087074	PPP1R15A	Protein phosphatase 1 regulatory subunit 15A	2.370	1126.54	3.11E-18	1.68E-15
31	ENSG00000160888	IER2	Immediate early response 2	2.367	2690.10	9.51E-22	1.12E-18
32	ENSG00000078804	TP53INP2	Tumor protein p53 inducible nuclear protein 2	2.348	443.02	1.60E-14	4.74E-12
33	ENSG00000116741	RGS2	Regulator of G-protein signaling 2	2.275	46.88	1.65E-06	9.38E-05
34	ENSG00000059804	SLC2A3	Solute carrier family 2 member 3	2.250	22.25	0.0001543	0.00382569
35	ENSG00000205869	KRTAP5-1	Keratin associated protein 5-1	2.177	32.04	1.94E-05	0.000723168
36	ENSG00000188396	TCTEX1D4	Tctex1 domain containing 4	2.156	25.40	7.51E-05	0.002127266
37	ENSG00000130513	GDF15	Growth differentiation factor 15	2.138	779.74	9.24E-17	3.87E-14
38	ENSG00000173334	TRIB1	Tribbles pseudokinase 1	2.029	1518.06	1.96E-19	1.41E-16
39	ENSG00000261150	EPPK1	Epiplakin 1	1.977	887.62	2.81E-17	1.26E-14
40	ENSG00000167604	NFKBID	NFKB inhibitor delta	1.960	716.84	2.00E-16	7.64E-14
41	ENSG00000089692	LAG3	Lymphocyte activating 3	1.937	29.12	3.43E-05	0.001134305
42	ENSG00000117228	GBP1	Guanylate binding protein 1	1.930	53.83	6.33E-07	3.97E-05
43	ENSG00000211448	DIO2	Deiodinase	1.918	69.39	9.94E-08	7.51E-06
44	ENSG00000139289	PHLDA1	Pleckstrin homology like domain family A member 1	1.871	961.02	1.35E-17	6.51E-15
45	ENSG00000171223	JUNB	JunB proto-oncogene	1.859	1147.16	2.63E-18	1.48E-15
46	ENSG00000186352	ANKRD37	Ankyrin repeat domain 37	1.854	92.46	1.09E-08	1.01E-06
47	ENSG00000128016	ZFP36	ZFP36 ring finger protein	1.810	1150.12	2.57E-18	1.48E-15
48	ENSG00000127528	KLF2	Kruppel like factor 2	1.805	124.52	9.93E-10	1.13E-07

No.	Ensemble ID	Gene	Full gene name	logFC	F	P value	FDR
49	ENSG00000165029	ABCA1	ATP binding cassette subfamily A member 1	1.772	12.15	0.002508064	0.032913909
50	ENSG00000118503	TNFAIP3	TNF alpha induced protein 3	1.661	46.77	1.68E-06	9.46E-05
51	ENSG00000139278	GLIPR1	GLI pathogenesis related 1	1.656	58.73	3.39E-07	2.25E-05
52	ENSG00000146278	PNRC1	Proline rich nuclear receptor coactivator 1	1.634	507.75	4.65E-15	1.55E-12
53	ENSG00000130066	SAT1	Spermidine/spermine N1-acetyltransferase 1	1.631	647.72	5.06E-16	1.82E-13
54	ENSG00000171303	KCNK3	Potassium two pore domain channel subfamily K member 3	1.581	12.69	0.002108333	0.028863507
55	ENSG00000169744	LDB2	LIM domain binding 2	1.581	11.88	0.00273803	0.034946381
56	ENSG00000107968	MAP3K8	Mitogen-activated protein kinase kinase kinase 8	1.567	282.81	8.84E-13	2.01E-10
57	ENSG00000112309	B3GAT2	Beta-1	1.562	16.89	0.000608193	0.011418554
58	ENSG00000105219	CNTD2	Cyclin N-terminal domain containing 2	1.553	35.78	9.79E-06	0.000415596
59	ENSG00000184545	DUSP8	Dual specificity phosphatase 8	1.552	481.37	7.54E-15	2.39E-12
60	ENSG00000181856	SLC2A4	Solute carrier family 2 member 4	1.551	12.30	0.00238758	0.031652483
61	ENSG00000153487	ING1	Inhibitor of growth family member 1	1.523	645.22	5.24E-16	1.84E-13
62	ENSG00000136826	KLF4	Kruppel like factor 4	1.504	380.38	6.30E-14	1.67E-11
63	ENSG00000143384	MCL1	BCL2 family apoptosis regulator	1.477	1498.22	2.21E-19	1.44E-16
64	ENSG00000062716	VMP1	Vacuole membrane protein 1	1.467	933.88	1.76E-17	8.15E-15
65	ENSG00000004799	PDK4	Pyruvate dehydrogenase kinase 4	1.453	25.25	7.77E-05	0.002188453
66	ENSG00000140465	CYP1A1	Cytochrome P450 family 1 subfamily A member 1	1.433	20.52	0.000234777	0.005379579
67	ENSG00000140511	HAPLN3	Hyaluronan and proteoglycan link protein 3	1.398	10.72	0.004034738	0.046265944
68	ENSG00000153443	UBALD1	UBA like domain containing 1	1.375	601.58	9.93E-16	3.40E-13
69	ENSG00000125657	TNFSF9	Tumor necrosis factor superfamily member 9	1.365	50.34	1.01E-06	6.09E-05
70	ENSG00000188785	ZNF548	Zinc finger protein 548	1.357	128.48	7.68E-10	8.98E-08
71	ENSG00000111859	NEDD9	Neural precursor cell expressed	1.357	154.12	1.68E-10	2.28E-08
72	ENSG00000075426	FOSL2	FOS like 2	1.355	872.29	3.29E-17	1.43E-14
73	ENSG00000130775	THEMIS2	Thymocyte selection associated family member 2	1.347	10.83	0.003882568	0.045199217
74	ENSG00000168874	ATOH8	Atonal bHLH transcription factor 8	1.347	12.50	0.002239738	0.030122852
75	ENSG00000120875	DUSP4	Dual specificity phosphatase 4	1.339	737.22	1.55E-16	6.27E-14
76	ENSG00000171790	SLFNL1	Schlafen like 1	1.322	19.02	0.0003442	0.007379281
77	ENSG00000162891	IL20	Interleukin 20	1.314	83.81	2.36E-08	2.04E-06
78	ENSG00000129951	PLPPR3	Phospholipid phosphatase related 3	1.288	10.57	0.004251797	0.048117903
79	ENSG00000172602	RND1	Rho family GTPase 1	1.238	149.02	2.23E-10	2.87E-08
80	ENSG00000172061	LRRC15	Leucine rich repeat containing 15	1.232	37.14	7.72E-06	0.000344853
81	ENSG00000115844	DLX2	Distal-less homeobox 2	1.213	25.06	8.11E-05	0.002264916
82	ENSG00000137331	IER3	Immediate early response 3	1.198	442.79	1.60E-14	4.74E-12
83	ENSG00000165997	ARL5B	ADP ribosylation factor like GTPase 5B	1.161	500.27	5.32E-15	1.73E-12
84	ENSG00000188001	TPRG1	Tumor protein p63 regulated 1	1.149	15.21	0.000981316	0.016387215
85	ENSG00000124882	EREG	Epiregulin	1.147	19.05	0.000341547	0.00733452
86	ENSG00000130522	JUND	JunD proto-oncogene	1.145	167.30	8.41E-11	1.24E-08
87	ENSG00000159556	ISL2	ISL LIM homeobox 2	1.128	16.27	0.000722665	0.013022009
88	ENSG00000114541	FRMD4B	FERM domain containing 4B	1.121	215.82	9.44E-12	1.86E-09
89	ENSG00000158050	DUSP2	Dual specificity phosphatase 2	1.109	122.17	1.16E-09	1.30E-07
90	ENSG00000163660	CCNL1	Cyclin L1	1.104	664.54	4.00E-16	1.48E-13
91	ENSG00000134107	BHLHE40	Basic helix-loop-helix family member e40	1.080	434.20	1.92E-14	5.41E-12

No.	Ensemble ID	Gene	Full gene name	logFC	F	P value	FDR
92	ENSG00000171174	RBKS	Ribokinase	1.067	61.74	2.36E-07	1.63E-05
93	ENSG00000198576	ARC	Activity regulated cytoskeleton associated protein	1.066	48.32	1.34E-06	7.80E-05
94	ENSG00000221995	TIAF1	TGFB1-induced anti-apoptotic factor 1	1.034	32.48	1.78E-05	0.00068583
95	ENSG00000197019	SERTAD1	SERTA domain containing 1	1.034	295.21	6.04E-13	1.40E-10
96	ENSG00000204618	RNF39	Ring finger protein 39	1.025	41.99	3.47E-06	0.000177593
97	ENSG00000112658	SRF	Serum response factor	1.023	438.68	1.75E-14	5.04E-12
98	ENSG00000109321	AREG	Amphiregulin	1.021	223.29	7.02E-12	1.42E-09
99	ENSG00000055483	USP36	Ubiquitin specific peptidase 36	1.016	398.71	4.13E-14	1.12E-11
100	ENSG00000136997	MYC	V-myc avian myelocytomatosis viral oncogene homolog	1.009	471.45	9.10E-15	2.82E-12
101	ENSG00000019186	CYP24A1	Cytochrome P450 family 24 subfamily A member 1	1.007	189.60	2.89E-11	5.00E-09
102	ENSG00000163659	TIPARP	TCDD inducible poly(ADP-ribose) polymerase	1.005	189.04	2.96E-11	5.06E-09

60 min timepoint

No.	Ensemble ID	Gene	Full gene name	logFC	F	P value	FDR
1	ENSG00000119508	NR4A3	Nuclear receptor subfamily 4 group A member 3	7.788	5967.51	3.50E-27	1.14E-23
2	ENSG00000125740	FOSB	FosB proto-oncogene	7.689	5703.40	5.61E-27	1.22E-23
3	ENSG00000135625	EGR4	Early growth response 4	7.534	1894.86	5.27E-22	4.03E-19
4	ENSG00000122877	EGR2	Early growth response 2	7.157	2158.71	1.37E-22	1.27E-19
5	ENSG00000135346	CGA	Glycoprotein hormones	6.760	530.02	2.44E-16	4.47E-14
6	ENSG00000123358	NR4A1	Nuclear receptor subfamily 4 group A member 1	6.555	11619.72	3.35E-30	4.36E-26
7	ENSG00000164949	GEM	GTP binding protein overexpressed in skeletal muscle	5.796	2292.62	7.32E-23	7.94E-20
8	ENSG00000118523	CTGF	Connective tissue growth factor	5.778	730.19	9.50E-18	2.29E-15
9	ENSG00000162772	ATF3	Activating transcription factor 3	5.539	6484.61	1.47E-27	6.38E-24
10	ENSG00000102195	GPR50	G protein-coupled receptor 50	5.053	104.18	1.41E-09	6.95E-08
11	ENSG00000169429	CXCL8	C-X-C motif chemokine ligand 8	4.913	108.96	9.49E-10	4.88E-08
12	ENSG00000120738	EGR1	Early growth response 1	4.743	7080.65	5.88E-28	3.83E-24
13	ENSG00000159200	RCAN1	Regulator of calcineurin 1	4.444	2270.75	8.08E-23	8.09E-20
14	ENSG00000185022	MAFF	MAF bZIP transcription factor F	4.327	1490.15	6.31E-21	3.04E-18
15	ENSG00000198732	SMOC1	SPARC related modular calcium binding 1	4.203	213.29	1.92E-12	1.70E-10
16	ENSG00000170345	FOS	Fos proto-oncogene	4.131	4937.21	2.52E-26	4.44E-23
17	ENSG00000222009	BTBD19	BTB domain containing 19	4.005	660.74	2.62E-17	5.51E-15
18	ENSG00000118503	TNFAIP3	TNF alpha induced protein 3	3.878	558.92	1.43E-16	2.69E-14
19	ENSG00000185950	IRS2	Insulin receptor substrate 2	3.530	1229.22	4.59E-20	1.87E-17
20	ENSG00000177606	JUN	Jun proto-oncogene	3.469	5738.75	5.26E-27	1.22E-23
21	ENSG00000179094	PER1	Period circadian clock 1	3.324	1681.84	1.81E-21	1.18E-18
22	ENSG00000188396	TCTEX1D4	Tctex1 domain containing 4	3.249	72.69	3.02E-08	1.07E-06
23	ENSG00000175592	FOSL1	FOS like 1	3.233	412.83	2.99E-15	4.69E-13
24	ENSG00000153234	NR4A2	Nuclear receptor subfamily 4 group A member 2	3.219	4495.31	6.69E-26	9.68E-23
25	ENSG00000205085	FAM71F2	Family with sequence similarity 71 member F2	3.081	23.22	9.31E-05	0.001039632
26	ENSG00000140465	CYP1A1	Cytochrome P450 family 1 subfamily A member 1	2.995	163.09	2.42E-11	1.81E-09
27	ENSG00000108551	RASD1	Ras related dexamethasone induced 1	2.910	1655.06	2.13E-21	1.32E-18
28	ENSG00000087074	PPP1R15A	Protein phosphatase 1 regulatory subunit 15A	2.906	1631.09	2.48E-21	1.47E-18

No.	Ensemble ID	Gene	Full gene name	logFC	F	P value	FDR
29	ENSG00000116741	RGS2	Regulator of G-protein signaling 2	2.813	83.77	9.24E-09	3.81E-07
30	ENSG00000205869	KRTAP5-1	Keratin associated protein 5-1	2.796	68.39	4.97E-08	1.64E-06
31	ENSG00000078804	TP53INP2	Tumor protein p53 inducible nuclear protein 2	2.782	718.52	1.12E-17	2.60E-15
32	ENSG00000179388	EGR3	Early growth response 3	2.749	4899.94	2.73E-26	4.44E-23
33	ENSG00000139178	C1RL	Complement C1r subcomponent like	2.745	17.11	0.000472338	0.003953361
34	ENSG00000120129	DUSP1	Dual specificity phosphatase 1	2.728	974.21	5.00E-19	1.86E-16
35	ENSG00000142871	CYR61	Cysteine rich angiogenic inducer 61	2.723	1205.70	5.60E-20	2.21E-17
36	ENSG00000171522	PTGER4	Prostaglandin E receptor 4	2.710	61.78	1.12E-07	3.34E-06
37	ENSG00000023445	BIRC3	Baculoviral IAP repeat containing 3	2.667	32.62	1.16E-05	0.000178475
38	ENSG00000173334	TRIB1	Tribbles pseudokinase 1	2.644	2512.40	2.83E-23	3.35E-20
39	ENSG00000136826	KLF4	Kruppel like factor 4	2.632	1361.40	1.60E-20	7.45E-18
40	ENSG00000167874	TMEM88	Transmembrane protein 88	2.629	162.66	2.48E-11	1.85E-09
41	ENSG00000211448	DIO2	Deiodinase	2.561	165.33	2.13E-11	1.64E-09
42	ENSG00000144655	CSRNP1	Cysteine and serine rich nuclear protein 1	2.542	2060.70	2.21E-22	1.92E-19
43	ENSG00000128342	LIF	Leukemia inhibitory factor	2.503	782.89	4.67E-18	1.34E-15
44	ENSG00000139289	PHLDA1	Pleckstrin homology like domain family A member 1	2.498	1877.37	5.80E-22	4.19E-19
45	ENSG00000115844	DLX2	Distal-less homeobox 2	2.462	138.91	1.07E-10	6.87E-09
46	ENSG00000198576	ARC	Activity regulated cytoskeleton associated protein	2.226	300.34	6.96E-14	8.31E-12
47	ENSG00000104856	RELB	RELB proto-oncogene	2.209	103.55	1.49E-09	7.30E-08
48	ENSG00000261150	EPPK1	Epiplakin 1	2.202	1942.38	4.08E-22	3.32E-19
49	ENSG00000180353	HCLS1	Hematopoietic cell-specific Lyn substrate 1	2.169	24.02	7.65E-05	0.000883787
50	ENSG00000127528	KLF2	Kruppel like factor 2	2.167	240.90	5.96E-13	5.88E-11
51	ENSG00000142178	SIK1	Salt inducible kinase 1	2.145	16.99	0.000488226	0.004060231
52	ENSG00000130775	THEMIS2	Thymocyte selection associated family member 2	2.143	33.74	9.28E-06	0.000149087
53	ENSG00000125787	GNRH2	Gonadotropin releasing hormone 2	2.101	22.10	0.000122852	0.001309519
54	ENSG00000164761	TNFRSF11B	TNF receptor superfamily member 11b	2.086	102.53	1.62E-09	7.85E-08
55	ENSG00000160888	IER2	Immediate early response 2	2.047	1618.78	2.68E-21	1.52E-18
56	ENSG00000185668	POU3F1	POU class 3 homeobox 1	2.007	14.94	0.000902094	0.006716678
57	ENSG00000197019	SERTAD1	SERTA domain containing 1	1.995	1189.94	6.41E-20	2.45E-17
58	ENSG00000147509	RGS20	Regulator of G-protein signaling 20	1.995	20.22	0.000200182	0.001958924
59	ENSG00000165029	ABCA1	ATP binding cassette subfamily A member 1	1.977	15.81	0.000691413	0.005369179
60	ENSG00000130066	SAT1	Spermidine/spermine N1-acetyltransferase 1	1.953	1510.06	5.50E-21	2.75E-18
61	ENSG00000117228	GBP1	Guanylate binding protein 1	1.944	44.53	1.35E-06	2.92E-05
62	ENSG00000143507	DUSP10	Dual specificity phosphatase 10	1.942	69.08	4.58E-08	1.52E-06
63	ENSG00000172738	TMEM217	Transmembrane protein 217	1.937	15.58	0.000742599	0.005708754
64	ENSG00000089692	LAG3	Lymphocyte activating 3	1.915	30.39	1.83E-05	0.000263604
65	ENSG00000055483	USP36	Ubiquitin specific peptidase 36	1.911	1769.98	1.07E-21	7.30E-19
66	ENSG00000130513	GDF15	Growth differentiation factor 15	1.902	697.76	1.51E-17	3.38E-15
67	ENSG00000167604	NFKBID	NFKB inhibitor delta	1.894	748.04	7.43E-18	1.90E-15
68	ENSG00000146278	PNRC1	Proline rich nuclear receptor coactivator 1	1.892	771.36	5.43E-18	1.50E-15
69	ENSG00000164251	F2RL1	F2R like trypsin receptor 1	1.879	613.79	5.54E-17	1.09E-14
70	ENSG00000111012	CYP27B1	Cytochrome P450 family 27 subfamily B member 1	1.836	286.72	1.10E-13	1.22E-11
71	ENSG00000138271	GPR87	G protein-coupled receptor 87	1.829	237.51	6.83E-13	6.64E-11

No.	Ensemble ID	Gene	Full gene name	logFC	F	P value	FDR
72	ENSG00000186352	ANKRD37	Ankyrin repeat domain 37	1.827	91.39	4.40E-09	1.92E-07
73	ENSG00000019549	SNAI2	Snail family transcriptional repressor 2	1.815	26.98	3.84E-05	0.000499351
74	ENSG00000075426	FOSL2	FOS like 2	1.814	1539.65	4.50E-21	2.44E-18
75	ENSG00000171873	ADRA1D	Adrenoceptor alpha 1D	1.805	17.28	0.000450256	0.003805248
76	ENSG00000051523	CYBA	Cytochrome b-245 alpha chain	1.797	83.81	9.21E-09	3.81E-07
77	ENSG00000187726	DNAJB13	DnaJ heat shock protein family (Hsp40) member B13	1.792	16.03	0.000647959	0.005092506
78	ENSG00000113070	HBEGF	Heparin binding EGF like growth factor	1.782	134.94	1.39E-10	8.70E-09
79	ENSG00000139278	GLIPR1	GLI pathogenesis related 1	1.754	55.82	2.48E-07	6.68E-06
80	ENSG00000143384	MCL1	BCL2 family apoptosis regulator	1.751	2674.04	1.48E-23	1.93E-20
81	ENSG00000168874	ATOH8	Atonal bHLH transcription factor 8	1.726	19.63	0.00023403	0.002233069
82	ENSG00000144063	MALL	Mal	1.723	60.30	1.36E-07	3.96E-06
83	ENSG00000086544	ITPKC	Inositol-trisphosphate 3-kinase C	1.719	897.27	1.16E-18	3.97E-16
84	ENSG00000111859	NEDD9	Neural precursor cell expressed	1.718	288.65	1.03E-13	1.15E-11
85	ENSG00000169548	ZNF280A	Zinc finger protein 280A	1.700	12.03	0.002304756	0.014169295
86	ENSG00000165917	RAPSN	Receptor associated protein of the synapse	1.700	12.38	0.002049834	0.012938209
87	ENSG00000138347	MYPN	Myopalladin	1.644	20.75	0.000173684	0.00174275
88	ENSG00000107968	MAP3K8	Mitogen-activated protein kinase kinase kinase 8	1.633	349.21	1.58E-14	2.16E-12
89	ENSG00000059728	MXD1	MAX dimerization protein 1	1.632	820.57	2.89E-18	8.75E-16
90	ENSG00000100906	NFKBIA	NFKB inhibitor alpha	1.632	1513.35	5.38E-21	2.75E-18
91	ENSG00000124882	EREG	Epiregulin	1.630	47.72	8.13E-07	1.88E-05
92	ENSG00000184545	DUSP8	Dual specificity phosphatase 8	1.626	569.31	1.19E-16	2.27E-14
93	ENSG00000184270	HIST2H2AB	Histone cluster 2 H2A family member b	1.615	11.66	0.002619566	0.015617798
94	ENSG00000155090	KLF10	Kruppel like factor 10	1.590	971.30	5.15E-19	1.86E-16
95	ENSG00000125657	TNFSF9	Tumor necrosis factor superfamily member 9	1.590	69.58	4.32E-08	1.45E-06
96	ENSG00000116285	ERRFI1	ERBB receptor feedback inhibitor 1	1.582	1296.82	2.64E-20	1.15E-17
97	ENSG00000205189	ZBTB10	Zinc finger and BTB domain containing 10	1.575	751.18	7.12E-18	1.85E-15
98	ENSG00000128590	DNAJB9	DnaJ heat shock protein family (Hsp40) member B9	1.572	413.45	2.95E-15	4.69E-13
99	ENSG00000168386	FILIP1L	Filamin A interacting protein 1 like	1.571	80.99	1.23E-08	4.90E-07
100	ENSG00000172602	RND1	Rho family GTPase 1	1.569	274.85	1.66E-13	1.74E-11
101	ENSG00000129749	CHRNA10	Cholinergic receptor nicotinic alpha 10 subunit	1.559	32.95	1.09E-05	0.000169195
102	ENSG00000172061	LRRC15	Leucine rich repeat containing 15	1.555	58.17	1.80E-07	4.99E-06
103	ENSG00000171223	JUNB	JunB proto-oncogene	1.552	828.51	2.62E-18	8.12E-16
104	ENSG00000153443	UBALD1	UBA like domain containing 1	1.539	868.60	1.62E-18	5.26E-16
105	ENSG00000129951	PLPPR3	Phospholipid phosphatase related 3	1.533	15.73	0.000708248	0.005473779
106	ENSG00000153487	ING1	Inhibitor of growth family member 1	1.532	650.77	3.06E-17	6.23E-15
107	ENSG00000128016	ZFP36	ZFP36 ring finger protein	1.532	723.54	1.04E-17	2.47E-15
108	ENSG00000163659	TIPARP	TCDD inducible poly(ADP-ribose) polymerase	1.520	669.34	2.30E-17	4.99E-15
109	ENSG00000188215	DCUN1D3	Defective in cullin neddylation 1 domain containing 3	1.516	327.98	2.93E-14	3.78E-12
110	ENSG00000130522	JUND	JunD proto-oncogene	1.495	355.25	1.33E-14	1.88E-12
111	ENSG00000114541	FRMD4B	FERM domain containing 4B	1.477	479.82	6.64E-16	1.14E-13
112	ENSG00000130164	LDLR	Low density lipoprotein receptor	1.467	1250.28	3.85E-20	1.62E-17
113	ENSG00000133639	BTG1	BTG anti-proliferation factor 1	1.464	447.35	1.34E-15	2.18E-13
114	ENSG00000198535	C2CD4A	C2 calcium dependent domain containing 4A	1.460	28.14	2.97E-05	0.000399817

No.	Ensemble ID	Gene	Full gene name	logFC	F	P value	FDR
115	ENSG00000105219	CNTD2	Cyclin N-terminal domain containing 2	1.411	23.56	8.56E-05	0.000971813
116	ENSG00000164463	CREBRF	CREB3 regulatory factor	1.406	103.48	1.50E-09	7.32E-08
117	ENSG00000100342	APOL1	Apolipoprotein L1	1.405	11.06	0.003220262	0.018414632
118	ENSG00000116717	GADD45A	Growth arrest and DNA damage inducible alpha	1.399	408.37	3.33E-15	5.16E-13
119	ENSG00000143367	TUFT1	Tuftelin 1	1.386	859.32	1.80E-18	5.73E-16
120	ENSG00000157557	ETS2	ETS proto-oncogene 2	1.375	734.79	8.91E-18	2.19E-15
121	ENSG00000132510	KDM6B	Lysine demethylase 6B	1.363	658.62	2.71E-17	5.60E-15
122	ENSG00000258947	TUBB3	Tubulin beta 3 class III	1.360	56.99	2.12E-07	5.77E-06
123	ENSG00000162461	SLC25A34	Solute carrier family 25 member 34	1.355	20.56	0.000182657	0.001812982
124	ENSG00000067082	KLF6	Kruppel like factor 6	1.352	292.26	9.10E-14	1.04E-11
125	ENSG00000004799	PDK4	Pyruvate dehydrogenase kinase 4	1.349	24.04	7.62E-05	0.000883214
126	ENSG00000109321	AREG	Amphiregulin	1.340	666.65	2.40E-17	5.11E-15
127	ENSG00000188785	ZNF548	Zinc finger protein 548	1.335	114.40	6.16E-10	3.31E-08
128	ENSG00000141682	PMAIP1	Phorbol-12-myristate-13-acetate-induced protein 1	1.333	233.80	7.95E-13	7.45E-11
129	ENSG00000140511	HAPLN3	Hyaluronan and proteoglycan link protein 3	1.321	7.95	0.010311978	0.045758742
130	ENSG00000105131	EPHX3	Epoxide hydrolase 3	1.320	9.03	0.006769616	0.033048985
131	ENSG00000179148	ALOXE3	Arachidonate lipoxygenase 3	1.318	27.42	3.48E-05	0.000457513
132	ENSG00000174010	KLHL15	Kelch like family member 15	1.317	344.87	1.78E-14	2.39E-12
133	ENSG00000141579	ZNF750	Zinc finger protein 750	1.307	63.38	9.16E-08	2.80E-06
134	ENSG00000185304	RGPD2	RANBP2-like and GRIP domain containing 2	1.305	46.68	9.58E-07	2.16E-05
135	ENSG00000163874	ZC3H12A	Zinc finger CCCH-type containing 12A	1.297	368.57	9.24E-15	1.34E-12
136	ENSG00000204386	NEU1	Neuraminidase 1	1.297	907.14	1.04E-18	3.65E-16
137	ENSG00000166833	NAV2	Neuron navigator 2	1.287	180.20	9.52E-12	7.64E-10
138	ENSG00000196428	TSC22D2	TSC22 domain family member 2	1.283	735.12	8.87E-18	2.19E-15
139	ENSG00000165997	ARL5B	ADP ribosylation factor like GTPase 5B	1.278	755.78	6.69E-18	1.81E-15
140	ENSG00000150782	IL18	Interleukin 18	1.277	41.23	2.35E-06	4.65E-05
141	ENSG00000134107	BHLHE40	Basic helix-loop-helix family member e40	1.275	709.98	1.26E-17	2.89E-15
142	ENSG00000062716	VMP1	Vacuole membrane protein 1	1.275	1308.26	2.41E-20	1.08E-17
143	ENSG00000006062	MAP3K14	Mitogen-activated protein kinase kinase kinase 14	1.273	186.40	6.92E-12	5.70E-10
144	ENSG00000182324	KCNJ14	Potassium voltage-gated channel subfamily J member 14	1.273	25.96	4.85E-05	0.000602136
145	ENSG00000183742	MACC1	MACC1	1.271	106.26	1.18E-09	5.88E-08
146	ENSG00000120875	DUSP4	Dual specificity phosphatase 4	1.263	796.58	3.91E-18	1.16E-15
147	ENSG00000152433	ZNF547	Zinc finger protein 547	1.262	26.81	3.99E-05	0.000513438
148	ENSG00000077150	NFKB2	Nuclear factor kappa B subunit 2	1.260	526.51	2.61E-16	4.72E-14
149	ENSG00000173846	PLK3	Polo like kinase 3	1.256	316.99	4.10E-14	5.13E-12
150	ENSG00000175197	DDIT3	DNA damage inducible transcript 3	1.255	131.33	1.78E-10	1.08E-08
151	ENSG00000153294	ADGRF4	Adhesion G protein-coupled receptor F4	1.248	78.11	1.66E-08	6.37E-07
152	ENSG00000107984	DKK1	Dickkopf WNT signaling pathway inhibitor 1	1.241	294.90	8.33E-14	9.59E-12
153	ENSG00000162975	KCNF1	Potassium voltage-gated channel modifier subfamily F member 1	1.231	119.41	4.20E-10	2.34E-08
154	ENSG00000162783	IER5	Immediate early response 5	1.225	883.92	1.35E-18	4.51E-16
155	ENSG00000145362	ANK2	Ankyrin 2	1.212	7.79	0.010984464	0.048135623
156	ENSG00000134531	EMP1	Epithelial membrane protein 1	1.205	7.85	0.010728064	0.047234693
157	ENSG00000171722	C1orf111	Chromosome 1 open reading frame 111	1.204	39.33	3.27E-06	6.28E-05

No.	Ensemble ID	Gene	Full gene name	logFC	F	P value	FDR
158	ENSG00000173110	HSPA6	Heat shock protein family A (Hsp70) member 6	1.203	9.28	0.006152349	0.030679244
159	ENSG00000167614	TTYH1	Tweety family member 1	1.203	10.94	0.0033632	0.019064483
160	ENSG00000171790	SLFNL1	Schlafen like 1	1.203	16.39	0.00058338	0.004666684
161	ENSG00000080007	DDX43	DEAD-box helicase 43	1.199	124.19	2.95E-10	1.74E-08
162	ENSG00000114757	PEX5L	Peroxisomal biogenesis factor 5 like	1.195	15.42	0.00077945	0.005925549
163	ENSG00000115641	FHL2	Four and a half LIM domains 2	1.185	136.38	1.26E-10	7.97E-09
164	ENSG00000187242	KRT12	Keratin 12	1.177	17.28	0.000450213	0.003805248
165	ENSG00000173559	NABP1	Nucleic acid binding protein 1	1.174	116.47	5.25E-10	2.87E-08
166	ENSG00000117525	F3	Coagulation factor III	1.174	31.74	1.39E-05	0.000209378
167	ENSG00000221995	TIAF1	TGFB1-induced anti-apoptotic factor 1	1.170	45.44	1.17E-06	2.58E-05
168	ENSG00000125347	IRF1	Interferon regulatory factor 1	1.161	137.97	1.14E-10	7.24E-09
169	ENSG00000119714	GPR68	G protein-coupled receptor 68	1.157	8.68	0.007740393	0.036596914
170	ENSG00000011478	QPCTL	Glutaminyl-peptide cyclotransferase like	1.154	268.72	2.06E-13	2.13E-11
171	ENSG00000165023	DIRAS2	DIRAS family GTPase 2	1.153	9.70	0.005254426	0.027051565
172	ENSG00000141458	NPC1	NPC intracellular cholesterol transporter 1	1.139	413.09	2.97E-15	4.69E-13
173	ENSG00000168646	AXIN2	Axin 2	1.139	48.42	7.30E-07	1.71E-05
174	ENSG00000137449	CPEB2	Cytoplasmic polyadenylation element binding protein 2	1.136	171.24	1.54E-11	1.21E-09
175	ENSG00000172183	ISG20	Interferon stimulated exonuclease gene 20	1.128	91.39	4.40E-09	1.92E-07
176	ENSG00000153714	LURAP1L	Leucine rich adaptor protein 1 like	1.122	271.86	1.84E-13	1.92E-11
177	ENSG00000112658	SRF	Serum response factor	1.115	628.86	4.33E-17	8.67E-15
178	ENSG00000185291	IL3RA	Interleukin 3 receptor subunit alpha	1.113	30.85	1.66E-05	0.00024247
179	ENSG00000013441	CLK1	CDC like kinase 1	1.106	499.26	4.45E-16	7.83E-14
180	ENSG00000132326	PER2	Period circadian clock 2	1.103	324.51	3.25E-14	4.15E-12
181	ENSG00000126500	FLRT1	Fibronectin leucine rich transmembrane protein 1	1.101	18.86	0.00028884	0.002639926
182	ENSG00000175505	CLCF1	Cardiotrophin-like cytokine factor 1	1.096	142.33	8.53E-11	5.70E-09
183	ENSG00000250423	KIAA1210	KIAA1210	1.092	14.66	0.000983147	0.007206989
184	ENSG00000105856	HBP1	HMG-box transcription factor 1	1.073	145.98	6.76E-11	4.63E-09
185	ENSG00000135114	OASL	2'-5'-oligoadenylate synthetase like	1.072	39.55	3.15E-06	6.06E-05
186	ENSG00000163660	CCNL1	Cyclin L1	1.060	751.74	7.06E-18	1.85E-15
187	ENSG00000171431	KRT20	Keratin 20	1.058	35.97	6.05E-06	0.000102761
188	ENSG00000167767	KRT80	Leratin 80	1.048	781.62	4.75E-18	1.34E-15
189	ENSG00000106546	AHR	Aryl hydrocarbon receptor	1.047	462.60	9.58E-16	1.60E-13
190	ENSG00000013588	GPRC5A	G protein-coupled receptor class C group 5 member A	1.045	329.49	2.80E-14	3.64E-12
191	ENSG00000138311	ZNF365	Zinc finger protein 365	1.045	32.43	1.20E-05	0.000184939
192	ENSG00000186086	NBPF6	Neuroblastoma breakpoint family member 6	1.044	16.71	0.000529922	0.004329528
193	ENSG00000159713	TPPP3	Tubulin polymerization promoting protein family member 3	1.040	8.71	0.007651531	0.036238965
194	ENSG00000241839	PLEKHO2	Pleckstrin homology domain containing O2	1.040	42.66	1.84E-06	3.82E-05
195	ENSG00000225828	FAM229A	Family with sequence similarity 229 member A	1.036	34.58	7.88E-06	0.000130124
196	ENSG00000053438	NNAT	Neuronatin	1.034	21.77	0.000133596	0.001403354
197	ENSG00000135048	TMEM2	Transmembrane protein 2	1.030	462.03	9.70E-16	1.60E-13
198	ENSG00000160678	S100A1	S100 calcium binding protein A1	1.030	50.43	5.38E-07	1.32E-05
199	ENSG00000181016	LSMEM1	Leucine rich single-pass membrane protein 1	1.028	24.42	6.96E-05	0.000817859
200	ENSG00000184697	CLDN6	Claudin 6	1.027	9.24	0.006237953	0.031031027

No.	Ensemble ID	Gene	Full gene name	logFC	F	P value	FDR
201	ENSG00000100234	TIMP3	TIMP metallopeptidase inhibitor 3	1.022	78.11	1.66E-08	6.37E-07
202	ENSG00000151014	NOCT	Nocturnin	1.017	263.98	2.46E-13	2.48E-11
203	ENSG00000108821	COL1A1	Collagen type I alpha 1 chain	1.017	110.41	8.44E-10	4.41E-08
204	ENSG00000253958	CLDN23	Claudin 23	1.016	11.73	0.002559698	0.015366451
205	ENSG00000198517	MAFK	MAF bZIP transcription factor K	1.012	341.77	1.95E-14	2.59E-12
206	ENSG00000111266	DUSP16	Dual specificity phosphatase 16	1.010	521.73	2.86E-16	5.10E-14
207	ENSG00000179133	C10orf67	Chromosome 10 open reading frame 67	1.006	14.37	0.001077506	0.007773696
208	ENSG00000117616	RSRP1	Arginine and serine rich protein 1	1.002	398.66	4.24E-15	6.41E-13

Supplementary Table S2: List of significantly downregulated differentially expressed genes obtained from RNA-sequencing of digitonin-injured MCF-7 breast cancer cells at 20-, 40- and 60-min post-injury.

20 min timepoint

No.	Ensemble ID	Gene	Full gene name	logFC	F	P value	FDR
1	ENSG00000128045	RASL11B	RAS like family 11 member B	-1.127	21.50	0.000229448	0.021369803

40 min timepoint

No.	Ensemble ID	Gene	Full gene name	logFC	F	P value	FDR
1	ENSG00000119938	PPP1R3C	Protein phosphatase 1 regulatory subunit 3C	-1.5182	337.94	1.82E-13	4.54E-11
2	ENSG00000132825	PPP1R3D	Protein phosphatase 1 regulatory subunit 3D	-1.0934	254.44	2.24E-12	4.94E-10
3	ENSG00000265972	TXNIP	Thioredoxin interacting protein [	-1.0129	212.38	1.08E-11	2.07E-09
4	ENSG00000168209	DDIT4	DNA damage inducible transcript 4	-1.2451	131.51	6.33E-10	7.54E-08
5	ENSG00000115507	OTX1	Orthodenticle homeobox 1	-1.0948	55.14	5.33E-07	3.43E-05
6	ENSG00000064195	DLX3	Distal-less homeobox 3	-1.0895	39.81	4.93E-06	0.000230281
7	ENSG00000168269	FOXI1	Forkhead box I1	-1.5154	39.65	5.06E-06	0.000234419
8	ENSG00000275793	RIMBP3	RIMS binding protein 3	-1.6442	29.96	2.90E-05	0.001013628
9	ENSG00000166333	ILK	Integrin linked kinase	-1.0922	15.86	0.000813568	0.014302941
10	ENSG00000182196	ARL6IP4	ADP ribosylation factor like GTPase 6 interacting protein 4	-1.0613	13.48	0.001644803	0.024269132
11	ENSG00000164056	SPRY1	Sprouty RTK signaling antagonist 1	-1.0933	12.77	0.002056024	0.02845443
12	ENSG00000182459	TEX19	Testis expressed 19	-1.4381	11.84	0.002773882	0.03515929

60 min timepoint

No.	Ensemble ID	Gene	Full gene name	logFC	F	P value	FDR
1	ENSG00000265972	TXNIP	Thioredoxin interacting protein	-1.494	394.34	4.72E-15	7.06E-13
2	ENSG00000119938	PPP1R3C	Protein phosphatase 1 regulatory subunit 3C	-1.268	218.93	1.50E-12	1.37E-10
3	ENSG00000203883	SOX18	SRY-box 18	-1.473	82.78	1.02E-08	4.16E-07
4	ENSG00000064195	DLX3	Distal-less homeobox 3	-1.541	81.32	1.19E-08	4.80E-07
5	ENSG00000180884	ZNF792	Zinc finger protein 792	-1.732	70.38	3.93E-08	1.35E-06
6	ENSG00000180739	S1PR5	Sphingosine-1-phosphate receptor 5	-1.054	67.37	5.61E-08	1.82E-06
7	ENSG00000115507	OTX1	Orthodenticle homeobox 1	-1.280	62.82	9.83E-08	2.96E-06
8	ENSG00000179023	KLHDC7A	Kelch domain containing 7A	-1.763	41.41	2.28E-06	4.53E-05
9	ENSG00000275793	RIMBP3	RIMS binding protein 3	-2.174	35.81	6.23E-06	0.000105583
10	ENSG00000165655	ZNF503	Zinc finger protein 503	-1.027	35.72	6.34E-06	0.000107102
11	ENSG00000168269	FOXI1	Forkhead box I1	-1.602	33.85	9.08E-06	0.000146315
12	ENSG00000187801	ZFP69B	ZFP69 zinc finger protein B	-1.158	26.32	4.46E-05	0.000561399
13	ENSG00000178150	ZNF114	Zinc finger protein 114	-1.027	25.88	4.93E-05	0.000610331
14	ENSG00000196544	BORCS6	BLOC-1 related complex subunit 6	-1.007	23.83	8.02E-05	0.000918341
15	ENSG00000158691	ZSCAN12	Zinc finger and SCAN domain containing 12	-1.049	22.47	0.000111951	0.001216649
16	ENSG00000197757	HOXC6	Homeobox C6	-1.224	21.24	0.000153201	0.001565621
17	ENSG00000165821	SALL2	Spalt like transcription factor 2	-1.008	16.97	0.000491928	0.00408319
18	ENSG00000187808	SOWAHD	Sosondowah ankyrin repeat domain family member D	-1.316	16.80	0.000516224	0.004246936
19	ENSG00000184507	NUTM1	NUT midline carcinoma family member 1	-2.121	15.52	0.0007558	0.005779517
20	ENSG00000203952	CCDC160	Coiled-coil domain containing 160	-1.425	13.96	0.001223006	0.008604016
21	ENSG00000132481	TRIM47	Tripartite motif containing 47	-1.147	13.40	0.001468096	0.00989738

No.	Ensemble ID	Gene	Full gene name	logFC	F	P value	FDR
22	ENSG00000164056	SPRY1	Sprouty RTK signaling antagonist 1	-1.207	11.93	0.002389462	0.014593661
23	ENSG00000234409	CCDC188	Coiled-coil domain containing 188	-1.729	11.76	0.0025297	0.015242611
24	ENSG00000173546	CSPG4	Chondroitin sulfate proteoglycan 4	-1.095	10.97	0.003323236	0.018912899
25	ENSG00000137843	PAK6	p21 (RAC1) activated kinase 6	-1.457	10.07	0.004590236	0.024285333
26	ENSG00000106006	HOXA6	Homeobox A6	-1.384	9.81	0.005045448	0.026193263
27	ENSG00000123405	NFE2	Nuclear factor erythroid 2	-1.101	9.69	0.005287014	0.027165608
28	ENSG00000171488	LRRC8C	Leucine rich repeat containing 8 family member C	-1.044	7.77	0.011065961	0.048394989

Supplementary Table S3: Biological processes (GO terms) significantly associated to the transcriptomic profile post-injury

Gene ontology analysis was performed using only differentially expressed transcripts were considered. For each associated GO term the total number of genes that are annotated with that specific term is denoted (under "Annotated"), as well as the number of significantly differentially expressed genes in the datasets that are associated with that term (under "Significant"). The "Expected" column refers the number of genes that would be expected to be significantly associated to each term by random chance. Finally, the p-value and FDR from Fisher's Exact Test reflect the probability of the association of significant transcriptomic changes with that particular GO term.

	**Häger *et al.***					**Rysä *et al.*, 2018**					**Wales *et al.*, 2016**				
	Annotated	Significant	Expected	Fisher	FDR	Annotated	Significant	Expected	Fisher	FDR	Annotated	Significant	Expected	Fisher	FDR
Cellular response to corticotropin-releasing hormone stimulus	5	3	0.06	1.50E-05	3.75E-05	5	3	0.02	6.00E-07	6.00E-06	5	3	0,05	7,20E-06	1,80E-05
Regulation of type B pancreatic cell proliferation	10	3	0.12	1.80E-04	2.84E-04	10	3	0.04	7.10E-06	3.04E-05	10	3	0,09	8,30E-05	1,31E-04
Positive regulation of smooth muscle cell proliferation	72	6	0.84	1.90E-04	2.85E-04	72	5	0.29	9.80E-06	3.67E-05	37	4	0,33	3,40E-04	3,40E-04
Fat cell differentiation	205	14	2.38	1.20E-07	7.20E-07	203	8	0.81	1.40E-06	8.40E-06	203	12	1,84	3,20E-07	1,60E-06
Response to cAMP	95	10	1.11	1.50E-07	7.50E-07	95	4	0.38	5.60E-04	6.00E-04	95	9	0,86	1,90E-07	1,14E-06
Negative regulation of ERK1 and ERK2 cascade	55	8	0.64	2.20E-07	8.25E-07						55	7	0.05	6.00E-07	2.57E-06
Response to hydrogen peroxide	126	10	1.47	2.10E-06	7.00E-06						126	13	1.14	1.10E-10	3.30E-09
Inactivation of MAPK activity	26	5	0.03	1.10E-05	3.00E-05						26	5	0.24	3.20E-06	1.07E-05
Skin morphogenesis	9	3	0.01	1.20E-04	2.12E-04						9	3	0.08	5.80E-05	1.02E-04
Inflammatory response	642	21	7.47	1.90E-05	4.38E+09						646	17	5.84	7.60E-05	1.27E-04
Cellular response to calcium ion	51	5	0.59	3.10E-04	4.04E-04						51	5	0.46	9.50E-05	1.43E-04
Circadian regulation of gene expression	54	6	0.63	3.70E-05	7.93E-05						54	5	0.49	1.30E-04	1.63E-04
Cellular response to cycloheximide	3	2	0.03	4.00E-04	4.10E-04						3	2	0.03	2.40E-04	2.57E-04
